# From Phineas Gage and Monsieur Leborgne to H.M.: Revisiting Disconnection Syndromes

**DOI:** 10.1093/cercor/bhv173

**Published:** 2015-08-12

**Authors:** M. Thiebaut de Schotten, F. Dell'Acqua, P. Ratiu, A. Leslie, H. Howells, E. Cabanis, M. T. Iba-Zizen, O. Plaisant, A. Simmons, N. F. Dronkers, S. Corkin, M. Catani

**Affiliations:** 1 Natbrainlab, Department of FANS, Institute of Psychiatry, Psychology and Neuroscience and; 2 Department of Neuroimaging, Institute of Psychiatry, King's College London, London, UK; 3 Brain Connectivity and Behaviour, Brain and Spine Institute, Paris, France; 4 Inserm U 1127; UPMC-Paris6, UMR_S 1127; CNRS UMR 7225, CRICM, GH Pitié-Salpêtrière, 75013 Paris, France; 5 Centre Hospitalier National d'Ophtalmologie des Quinze-Vingts, Paris, France; 6 University of Paris-Descartes, GH Pitié-Salpêtrière, URDIA, EA4465, Paris, France; 7 VA Northern California Health Care System, Martinez, CA, USA; 8 Department of Neurology, University of California, Davis, CA, USA; 9 National Research University Higher School of Economics, Russian Federation; 10 Department of Brain and Cognitive Sciences, Massachusetts Institute of Technology, Cambridge, MA, USA

**Keywords:** behavioral neurology, brain lesion, diaschisis, disconnection syndromes, white matter

## Abstract

On the 50th anniversary of Norman Geschwind's seminal paper entitled ‘Disconnexion syndrome in animal and man’, we pay tribute to his ideas by applying contemporary tractography methods to understand white matter disconnection in 3 classic cases that made history in behavioral neurology. We first documented the locus and extent of the brain lesion from the computerized tomography of Phineas Gage's skull and the magnetic resonance images of Louis Victor Leborgne's brain, Broca's first patient, and Henry Gustave Molaison. We then applied the reconstructed lesions to an atlas of white matter connections obtained from diffusion tractography of 129 healthy adults. Our results showed that in all 3 patients, disruption extended to connections projecting to areas distant from the lesion. We confirmed that the damaged tracts link areas that in contemporary neuroscience are considered functionally engaged for tasks related to emotion and decision-making (Gage), language production (Leborgne), and declarative memory (Molaison). Our findings suggest that even historic cases should be reappraised within a disconnection framework whose principles were plainly established by the associationist schools in the last 2 centuries.

## Introduction

Much of our knowledge about higher cognitive functions and complex behaviors derives from the description of historic seminal cases that helped shape neuroscience (Finger 1994; Compston 2009, 2011a,b, 2014). These cases reinforced localizationist ideas of cognitive functions related to the activity of discrete and fairly localized brain regions (Catani, Dell'acqua, Bizzi et al. 2012), including the association of social behavior with orbitofrontal cortex (Harlow 1868; Damasio et al. 1994), speech production with Broca's area (Broca 1861a,b), and declarative memory with medial temporal lobe structures (Scoville and Milner 1957).

Localization by area is an oversimplification of the actual workings of the brain (Damasio and Damasio 1989; Mesulam 1999; Catani, Dell'acqua, Bizzi et al. 2012). The localizationist bias stems from 2 main limitations. First, the overall idea of equating localization of symptoms with localization of functions may be incorrect. This point was already raised by several authors defending associationist theories, who argued that it is entirely possible that some symptoms can be explained by a secondary effect on other regions distant from the site of the damage but functionally impaired (Wernicke 1874; Jackson 1881; Lichtheim 1885; Jackson 1894; Dejerine 1895; Monakow 1914). Second, for a long time we have been unable to map lesions onto discrete circuits due to a lack of methods for visualizing single tracts in the living human brain (Catani and ffytche 2005). Indeed, modern neuroimaging has shown that many complex functions rely on the coordinated activity of distant regions connected by long-range fibers coursing through the cerebral white matter. Damage either to cortical areas or to underlying connections has far-reaching consequences on distant regions (Baron et al. 1981) through either diaschisis (i.e., dysfunction of a distant region connected to the damaged area) (Monakow 1897, 1914; Carrera and Tononi 2014) or disconnection (i.e., dysfunction of 2 intact areas connected by a damaged tract) (Wernicke 1874; Geschwind 1965a,b; Catani and ffytche 2005). However, the understanding of neuroanatomy during the first half of the 20th century was insufficient to capture the complexity of psychological functions. For many researchers, anatomy became largely irrelevant to the development of psychological models of function and dysfunction (Marie 1906; Brodmann 1909; Head 1926; Lashley 1929). For others, detailed cortical parcellation and localization became the only way to understanding cognitive functions (Von Economo and Koskinas 1925; Vogt and Vogt 1926).

Inspired by this vivid debate between localizationism and holism and motivated by the recent reports on the behavioral manifestations in animals with interhemipheric disconnection (Myers and Sperry 1953; Schrier and Sperry 1959; Gazzaniga et al. 1963), Norman Geschwind reappraised the associationist ideas in his Brain paper entitled “Disconnexion syndromes in animals and man”:

In the pages which follow I hope to give an account of the implications of thinking in terms of disconnexions for both clinical practice and research. The synthesis presented here was developed piecemeal out of study of the literature and clinical observation. I will not, however, present it in the order of its development but rather will try to organize the facts and theories along simple anatomical lines. There is, I believe, a unity in the theory which justifies this approach, and I hope that it will significantly contribute to clarification of the presentation. There are many facts recorded in the following pages; there is also much speculation which is, however, nearly all subject to the checks of future experiment and clinical observation.’ (Geschwind 1965a,b)

In his 2-part paper, Geschwind proposed a way of thinking that would influence future generations. The “facts recorded”, to which he refers, came mainly from 2 sources: 1) data on the anatomy of connections derived, when possible, from pioneering primate studies conducted during the middle of the 20th century (Glees et al. 1950; Adey and Meyer 1952; Nauta 1964) and 2) clinical observation of patients who underwent postmortem autopsy examinations (Geschwind and Kaplan 1962). The merit of his approach was the aim to bridge the gap between these 2 fields in a pre-imaging era. Well aware of the limitations of this approach, he acknowledged that his speculations were to be verified by future experiments and clinical observation. This test had to wait another 10 years before early PET, SPECT, and CT scanners became available for clinical anatomical correlation studies (Gainotti et al. 1972; Naeser et al. 1982). Patients were studied with extensive neuropsychological batteries of tests to document their symptoms in detail, coupled with lesion mapping and group analysis (Damasio and Damasio 1989). Both cortical and subcortical lesion localization were considered a crucial contribution to the clinical presentation. Thanks to Geschwind's vision, the anatomy of white matter connections derived from 19th century postmortem dissections was revisited in the living human brain and disconnection lesions to specific tracts were considered a valid mechanism for newly described syndromes (e.g., tactile agnosia; Geschwind and Kaplan 1962). Geschwind's premature death in 1984 robbed him of the opportunity to appreciate the tremendous impact that his ideas, coupled with methodological advancements in the field of white matter imaging, had on contemporary behavioral neurology. This advance is particularly striking when we consider the development of diffusion MRI, which provides unprecedented access to the anatomy of white matter pathways in the living human brain. One advantage of this approach is the possibility of mapping the anatomical trajectories of cortico-cortical and cortico-subcortical pathways and correlate anatomical variation with cognitive performance (Catani and Thiebaut de Schotten 2012; Rojkova et al. 2015). Tractography provides anatomical information about white matter organization, and functional correlates can be proposed through tractography, combined with other functional methods, or its application to brain-lesioned patients. For many historic patients with unique brain lesions, however, information on the extent of white matter damage is unavailable.

On the 50th anniversary of Geschwind's seminal contribution, we pay tribute to his work by revisiting the clinico-anatomical correlations of 3 famous neurological patients. For the first time, we combine advanced diffusion methods (Thiebaut de Schotten, Dell'Acqua et al. 2011; Catani, Dell'acqua, Vergani et al. 2012; Dell'Acqua and Catani 2012) and meta-analysis of functional MRI studies (Spaniol et al. 2009; Yarkoni et al. 2011) to propose damage to connections and explain symptoms using a network approach. The 2 complementary approaches allow identification of the overlap between the altered structural networks and the impaired functional networks in each patient. Our findings contribute to a better understanding of brain networks and the effect of disconnection on classic neurobehavioral syndromes.

## Methods

In this study, we collected original datasets of 3 cases that represent true milestones in the history of neurology. The case of Phineas Gage described by John Harlow (1868) marked the beginning of modern clinical investigations of the frontal lobe and related behaviors (Mesulam 1990, 2002; Damasio et al. 1994; Ratiu and Talos 2004a,b). Louis Victor Leborgne, also known as Monsieur Leborgne or “tan tan,” was the first non-fluent aphasic patient reported by Paul Broca in 1861 (Dronkers et al. 2007; Domanski 2013). The case of Henry Gustave Molaison, known in the scientific community as H.M., has helped us to understand the link between medial temporal lobe damage and memory deficits (Corkin 2013). For these 3 cases, we were able to obtain digital data for their skull (Phineas Gage) or brain MRI (postmortem for Monsieur Leborgne and in vivo for H.M.). Below we give a brief account of these 3 cases and details on the datasets we obtained. We then explain how their lesions were reconstructed, the process of normalizing their data sets to a common space of reference, and the method of mapping their lesions onto an atlas of white matter connections from the tractography of 129 healthy subjects.

### Phineas Gage, Louis Victor Leborgne, and Henry Gustave Molaison: Clinical History and Imaging Processing

#### Phineas Gage (1823–1860)

Gage was 25 years old when he made a costly mistake at his workplace that resulted in an iron bar passing through the left side of his skull. Despite extensive damage to his forehead, he survived the accident, but not without consequences. According to John Harlow, the local doctor, who followed Gage throughout his recovery, he became “fitful, irreverent, indulging at times in the grossest profanities (which was not previously his custom), manifesting little deference for his fellows, impatient of restraint or advice when it conflicts with his desires.” In this regard, his mind was radically changed, so decidedly that his friends and acquaintances said he was “no longer Gage” (Harlow 1868). Harlow argued that the behavioral changes in Gage's personality were the direct result of the damage to the left frontal lobe. Unfortunately, there is no detailed psychological assessment of Gage at the time of the incident or at any point in the following years of his short life; but his clinical manifestations have been interpreted as resulting from deficits in rational decision-making and emotion processing (Damasio et al. 1994).

**Figure 1.**
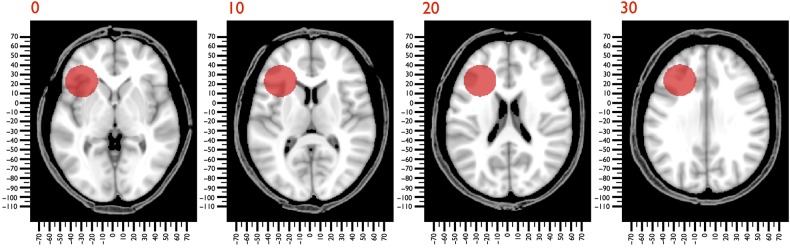
CAT scan of Gage's skull registered to MNI152 space. The trajectory of the bar through the skull is indicated in red.

After his death, Gage's skull and tamping iron have been housed in the Warren Museum of Anatomy in Boston. In our study, we used the axial (0.5 × 0.5 × 0.5 mm) computed tomography scan (CT-scan, Siemens AG, Erlangen, Germany) of Gage's skull acquired by Ratiu and Talos (2004a,b) and the dimensions of the original tamping iron to create a tridimensional model of the bar passing through Gage's skull (diameter 31.75 mm). The bar entered under the left zygomatic bone and passed through the greater and lesser wings of the sphenoid bone, creating the hole we observed near the midline of the skull in the left frontal bone. We then registered Gage's skull to the Montreal Neurological Institute (MNI) space (MNI152 nonlinear 6th generation; http://www.bic.mni.mcgill.ca) using affine and elastic deformation provided in the MIPAV v5.3.4 software package (http://mipav.cit.nih.gov) and the following anatomical landmarks: vertex, nasion, subnasal point, left and right supra-auricular point, maximum occipital point, lateral pterygoid plate, and external occipital protuberance. After registration, the simultaneous display of the trajectory of the bar and the MNI 152 brain enabled us to estimate the extent of the lesion (Fig. 1).

#### Louis Victor Leborgne (1809–1861)

**Figure 2.**
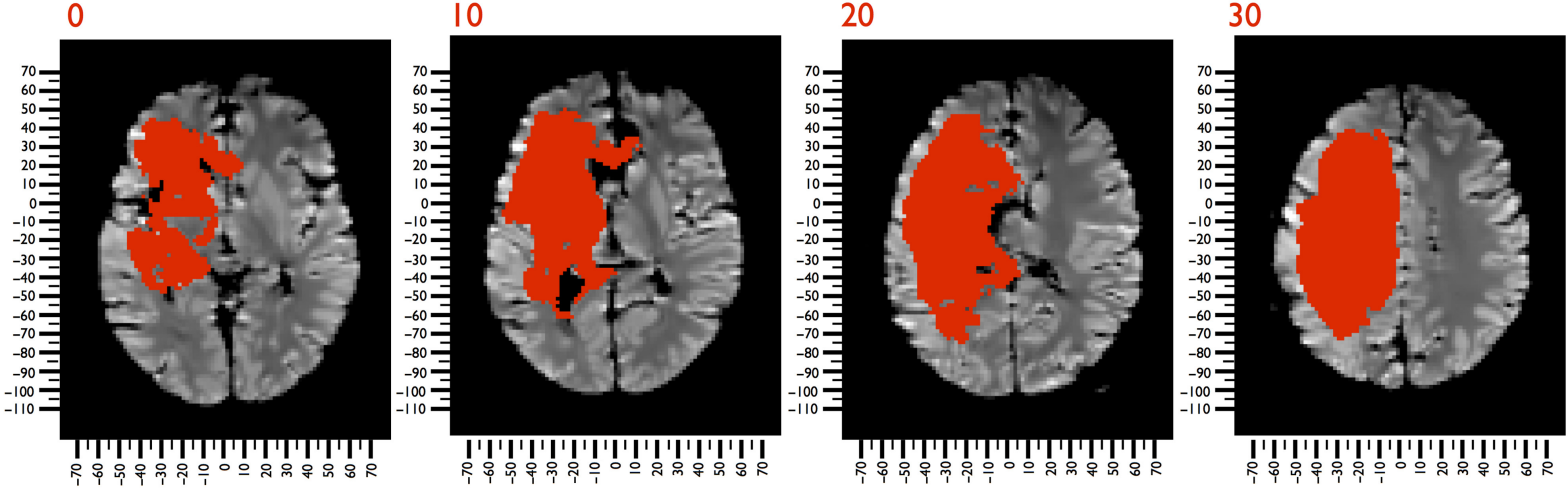
*T*_1_-weighted MRI images of Leborgne's brain, registered to MNI152 space. The damage produced by the stroke is indicated in red.

In 1839, Leborgne, a 30-year-old Frenchman, was admitted to Bicêtre Hospital following the sudden loss of his ability to speak. Leborgne was born in Morêt-sur-Loing and lost his mother at the age of 3. He moved to Paris with his family when he was 11 years old, and it is very likely that he received a formal education, his father being a teacher and all his siblings literate (Domanski 2013). He had epilepsy in his youth and lived at home until he became mute. At the time of his admission to the hospital, he was unmarried, and his father died shortly thereafter, which may explain why he remained an inmate at Bicêtre Hospital for 21 years. His condition eventually deteriorated, and he became paralyzed on his right side, spending the last 7 years of his life bedridden. In 1861, he developed gangrene in his right leg and was transferred to the surgical ward. Here, he was seen by the attending doctor, Paul Broca, who could do nothing to save his life. Broca had just returned from a meeting of the Société d'Anthropologie de Paris where Ernest Auburtin presented the case of Monsieur Cullerier, a patient who had shot himself in the head and was admitted to Saint-Louis hospital with an open wound to his forehead. Auburtin took this opportunity to test the hypothesis that speech was localized in the frontal lobe as suggested by his father in law, Jean-Baptiste Bouillaud, and by Franz Joseph Gall before him. He applied light pressure with a blade to the wounded man's frontal lobe, and his speech “suddenly terminated; a word that had been commenced was cut in two. The faculty of speech reappeared as soon as the compression ceased” (Auburtin 1861). Broca saw in Leborgne the opportunity to confirm at the autopsy table Aubertin's prediction about speech localization. Indeed, he found a lesion in the posterior third of the left inferior frontal gyrus (Fig. 2). Broca presented his work to the Société d'Anthropologie and published his findings the same year (Broca 1861a,b). Broca's report, although certainly not the first on the topic (see, Auburtin 1863 for a review of the cases reported before Broca), served as a signpost for the beginning of the modern study of cerebral localization.

Leborgne's brain has been preserved in the Dupuytren Museum in Paris for the past 150 years. In 2007, Dronkers and colleagues used a 1.5 T MRI scanner (General Electric Signa Echospeed HDX LCC Magnet 8.2.5) to acquire *T*_1_-weighted (1 × 1 × 1 mm) images of Leborgne's brain (Dronkers et al. 2007). With these images, it was possible to define Leborgne's lesion, using automated methods for lesion identification (ALI) (Seghier et al. 2008). ALI automatically classifies *T*_1_ maps into a set of probabilistic maps of gray matter, white matter, cerebrospinal fluid, and atypical tissue. The probability of damage refers to the likelihood that the damage occurred within a given voxel. If ALI identified a damaged tissue with a probability that equals 50%, there is a 50–50% chance that this tissue is damaged. Therefore, a voxel was classified as being damaged when >50% probability was detected (Fig. 2). Leborgne's brain was registered with the MNI152 using the affine and elastic deformation provided in the Statistical Parametric Mapping 8 software package (SPM8; http://www.fil.ion.ucl.ac.uk); a mask of the lesion was used to exclude the contribution of the damaged voxels to the registration (Friston et al. 1995; Brett et al. 2001).

#### Henry Gustave Molaison (1926–2008)

Molaison had petit mal seizures that began at age 10 and grand mal seizures that began at age 15. During adolescence, the epileptic attacks became more severe and were uncontrolled with pharmacological treatment (Mauguiere and Corkin 2015). His family doctor advised his parents to consult William Beecher Scoville, a neurosurgeon at the Hartford Hospital in Connecticut, USA. At that time, Scoville was performing psychosurgical procedures on patients with psychosis, consisting of the unilateral removal of medial temporal lobe structures. Scoville made the fortuitous observation that the operation was effective in reducing seizures in 2 psychotic women who had epilepsy (Scoville et al. 1953). Molaison's EEG results did not indicate an epileptic focus, but showed diffuse bilateral activity, on the basis of which Scoville decided to perform the experimental procedure in both left and right medial temporal lobes. The treatment palliated his seizures, but unexpectedly left him with a severe and lasting anterograde amnesia (Scoville and Milner 1957). This declarative memory impairment affected his ability to record new events and facts postoperatively. Molaison was able to maintain information online for about 30 s, but his ability to convert short-term memories into long-term memories was lost (Corkin 1984).

**Figure 3.**
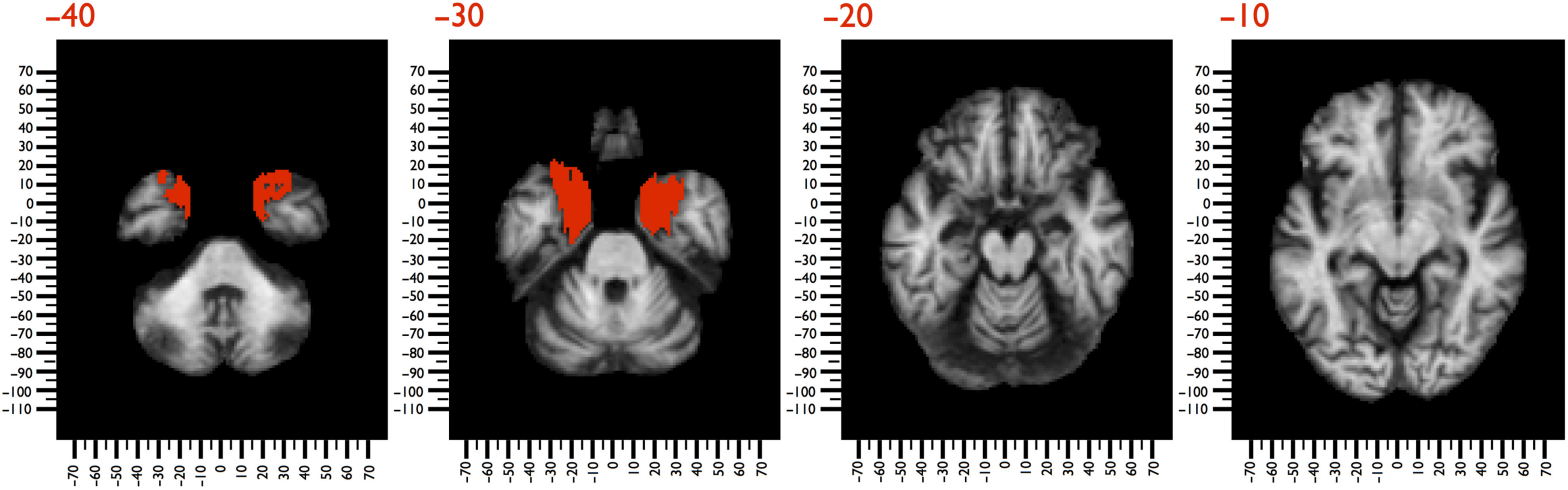
*T*_1_-weighted MRI images of Molaison's brain, registered to MNI152 space. The bilateral temporal damage produced by the surgery is shown in red.

In 1993, Corkin and colleagues collected *T*_1_-weighted MRI images (1 × 1 × 1 mm) of Molaison's brain using a 1.5 T scanner (General Electric Signa, Milwaukee, WI, USA) (details of the acquisition in Corkin et al. 1997). We used these images to define the lesions in Molaison's brain using ALI (Seghier et al. 2008) (Fig. 3). The lesion analysis and registration to the MNI152 were the same as for Leborgne's data set.

### Mapping Disconnections in Phineas Gage, Louis Victor Leborgne, and Henry Gustave Molaison

The next step in the analysis was to map lesions from each patient onto tractography reconstructions of white matter pathways obtained from a group of healthy controls. We first obtained diffusion data sets and tract reconstructions, then used complementary approaches to map the disconnections (tract specific vs. data-driven), and later conducted meta-analyses for each patient to validate the disconnection results with complementary fMRI activation studies published in the literature.

#### Diffusion-Weighted Imaging Acquisition

We recruited 129 healthy, right-handed volunteers (59 male, 70 female) aged 18–79 years and diffusion MRI scans were obtained from each participant. We acquired 60 contiguous near-axial slices on a 3T GE Signa HDx TwinSpeed system (General Electric, Milwaukee, WI, USA) with the following parameters: rostro-caudal phase encoding, voxel size 2.4 × 2.4 × 2.4 mm, matrix 128 × 128, slices 60, NEX 1, TE 93.4 ms, *b*-value 3000 s/mm^2^, 60 diffusion-weighted directions, and 7 non-diffusion-weighted volumes, using a spin-echo EPI sequence. Cardiac gating was applied with effective TR of 20/30 R–R intervals. At each slice, raw diffusion-weighted data were simultaneously registered and corrected for subject motion and geometrical distortions using Explore DTI (http://www.exploredti.com; Leemans and Jones 2009). Standard diffusion tensor tractography does not allow the reconstruction of the 3 branches of the superior longitudinal fasciculus (SLF I, II and III) because of the crossing of the dorsal association fibers with commissural and projection fibers (Thiebaut de Schotten, ffytche et al. 2011; Dell'Acqua and Catani 2012). Hence, we used spherical deconvolution to estimate multiple orientations in voxels containing crossing fibers and visualize the 3 branches of the SLF (Alexander 2006). A modified (damped) version of the Richardson-Lucy algorithm for spherical deconvolution (Dell'acqua et al. 2010) was employed using StarTrack software (http://www.natbrainlab.com) a freely available MATLAB 7.8 (http://www.mathworks.com) toolbox. Algorithm parameters were chosen as previously described (Dell'Acqua et al. 2013). A fixed fiber response corresponding to a shape factor of α = 1.5 × 10^−3^ mm^2^/s was chosen (Dell'Acqua et al. 2013). Fiber orientation estimates were obtained by selecting the orientation corresponding to the peaks (local maxima) of the fiber orientation distribution (FOD) profiles. To exclude spurious local maxima, we applied an absolute and a relative threshold. A first “absolute” threshold excluded small local maxima due to noise or isotropic tissue. This threshold was 3 times the amplitude of a spherical FOD obtained from a gray matter isotropic voxel. A second “relative” threshold of 8% of the maximum amplitude of the FOD was applied to remove the remaining local maxima with values greater than the absolute threshold (Dell'acqua et al. 2010).

#### Tractography

Whole brain tractography was performed from brain voxels with at least 1 fiber orientation. Streamlines were reconstructed using a modified Euler integration algorithm (Dell'Acqua et al. 2013). In regions with crossing white matter bundles, the algorithm followed the orientation vector of least curvature (Schmahmann et al. 2007). Streamlines were halted when a voxel without fiber orientation was reached or when the curvature between 2 steps exceeded a threshold of 45°.

#### Atlas-Based Analysis of Disconnection

**Figure 4.**
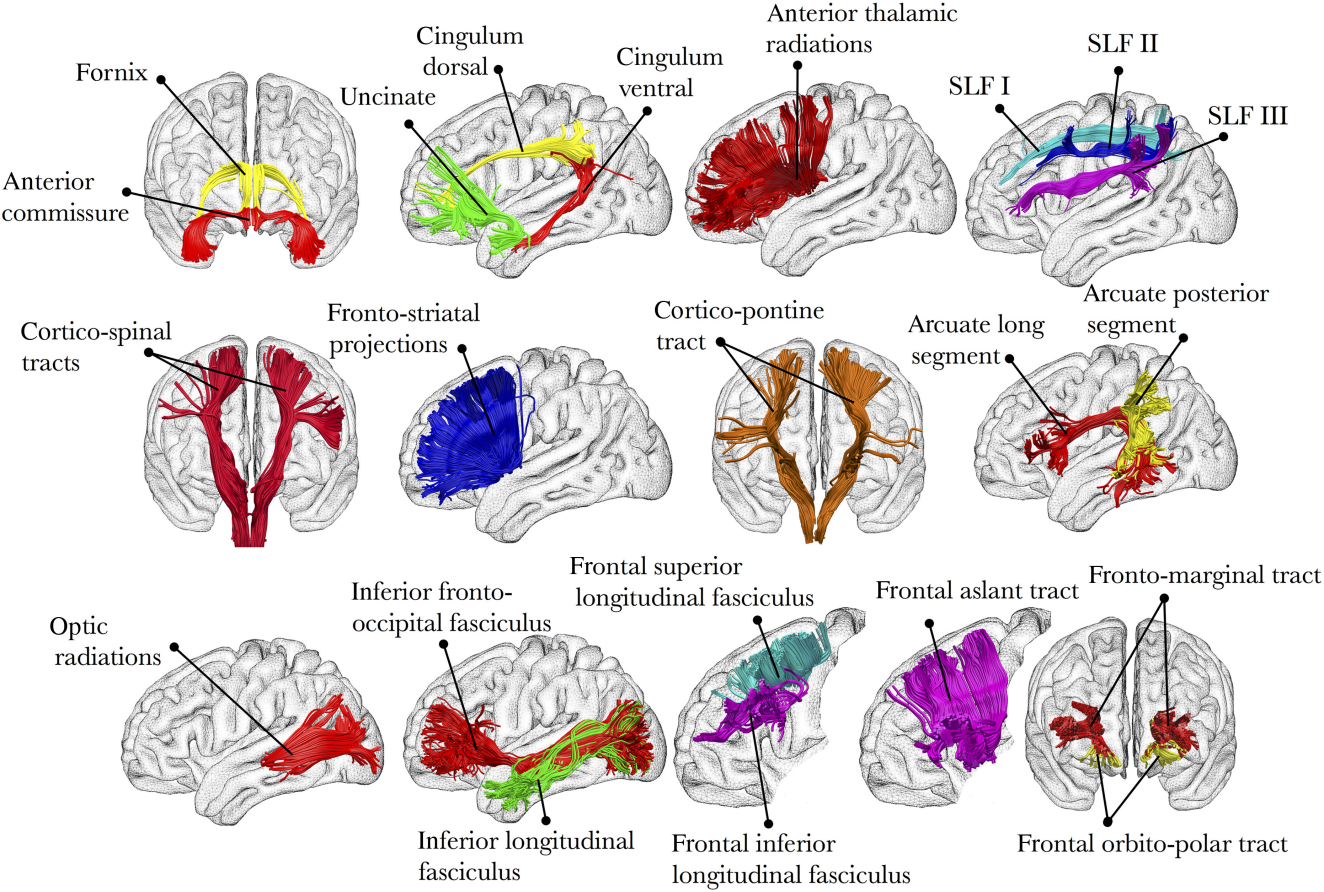
Summary of all the tracts included in our analysis.

For this analysis, we created an atlas of the human brain connections from the 129 healthy participants according to methods described in previous work (Thiebaut de Schotten, ffytche et al. 2011; Catani, Dell'acqua, Vergani et al. 2012; Catani and Thiebaut de Schotten 2012). Tractography dissection of the fornix, cingulum, uncinate fasciculus, inferior longitudinal fasciculus, and inferior fronto-occipital fasciculus was performed using a multiple region-of-interest (ROI) approach (Catani and Thiebaut de Schotten 2008). The anterior, posterior, and long segments of the arcuate fasciculus were dissected using a 2 ROI approach as described by Catani et al. (2007), and the cortico-spinal tract and optic radiations were dissected following the guidelines provided by Thiebaut de Schotten, ffytche and colleagues (2011). The anterior thalamic and fronto-striatal projections were reconstructed using previously published methods (Behrens et al. 2003; Cohen et al. 2009). We followed earlier reports in dissecting the frontal aslant tract, orbitopolar tracts, and frontal superior and inferior longitudinal fasciculi (Catani, Dell'acqua, Vergani et al. 2012; Thiebaut de Schotten et al. 2012). We isolated the 3 branches of the superior longitudinal fasciculus (SLF I, II, and III) using a multiple ROI approach (Thiebaut de Schotten, Dell'Acqua et al. 2011). In total, 22 tracts were reconstructed (see Fig. 4 for a visual summary of all these connections).

For each tract, binary visitation maps were created by assigning each voxel a value of 1 or 0, depending on whether the voxel was intersected by the streamlines of the tract. For each participant, convergence (CS) maps contrasting for white matter (Dell'acqua et al. 2006) were registered to the MNI152 template provided with the FMRIB Software Library package (FSL, http://www.fmrib.ox.ac.uk/fsl/). For the registration, we combined affine with diffeomorphic deformations (Avants et al. 2007; Klein et al. 2009) using Advance Normalization Tools (ANTs, http://www.picsl.upenn.edu/ANTS/). Binary visitation maps of each dissected tract were normalized to MNI space using both affine and diffeomorphic deformations. Normalized binary visitation maps were then averaged to create percentage overlap maps, and we used 50% overlap maps for the localization and quantification of the lesions (Thiebaut de Schotten et al. 2014). We quantified the severity of the disconnection by measuring the proportion of the tract disconnected (Thiebaut de Schotten et al. 2008) using Tractotron software (http://www.brainconnectivitybehaviour.eu). The severity of the disconnection was converted into *z*-values, allowing us to assess which tracts were statistically more damaged (*z* > 1.96; 2-tailed *P* < 0.05). All tracts were included in the *z*-score calculation.

#### Lesion-Based Approach to Mapping Disconnection

The inverse of the affine and diffeomorphic deformations calculated according to the description given above was used to register the normalized lesions of Gage, Leborgne, and Molaison to the native space of the 129 participants. In each healthy dataset, Tractotron software allowed us to register lesions as seedpoints to track streamlines passing through the damaged regions (http://www.brainconnectivitybehaviour.eu). We created a binary visitation map of the streamlines intersecting the lesion. These maps were normalized to MNI space using the affine and diffeomorphic deformations calculated above. We created percentage overlap maps by summing at each point in MNI space the normalized visitation map of each subject; hence, the value in each voxel of the visitation maps varied according to intersubject variability. These maps were projected onto the average 3D rendering of MNI152 using the Brainvisa 4.3 package (http://brainvisa.info) (Figs 6–8).

#### Meta-analyses

To identify the functional networks damaged in the 3 cases we applied a meta-analysis approach to functional MRI studies using a method described by Yarkoni et al. (2011; http://neurosynth.org) was used to identify the functional networks damaged in the 3 cases. We searched for brain regions that are consistently activated in studies that load highly on 3 features: “decision-making” (see Supplementary References 1) and “emotion” (see Supplementary References 2) for Gage and “fluency” (see Supplementary References 3) for Leborgne. For Molaison, we used a previously published meta-analysis, which reported areas separating activation related to encoding and retrieval of episodic memories (Spaniol et al. 2009). The results were superimposed on the 3D reconstruction of the MNI152 images (Figs 6–8).

## Results

**Figure 5.**
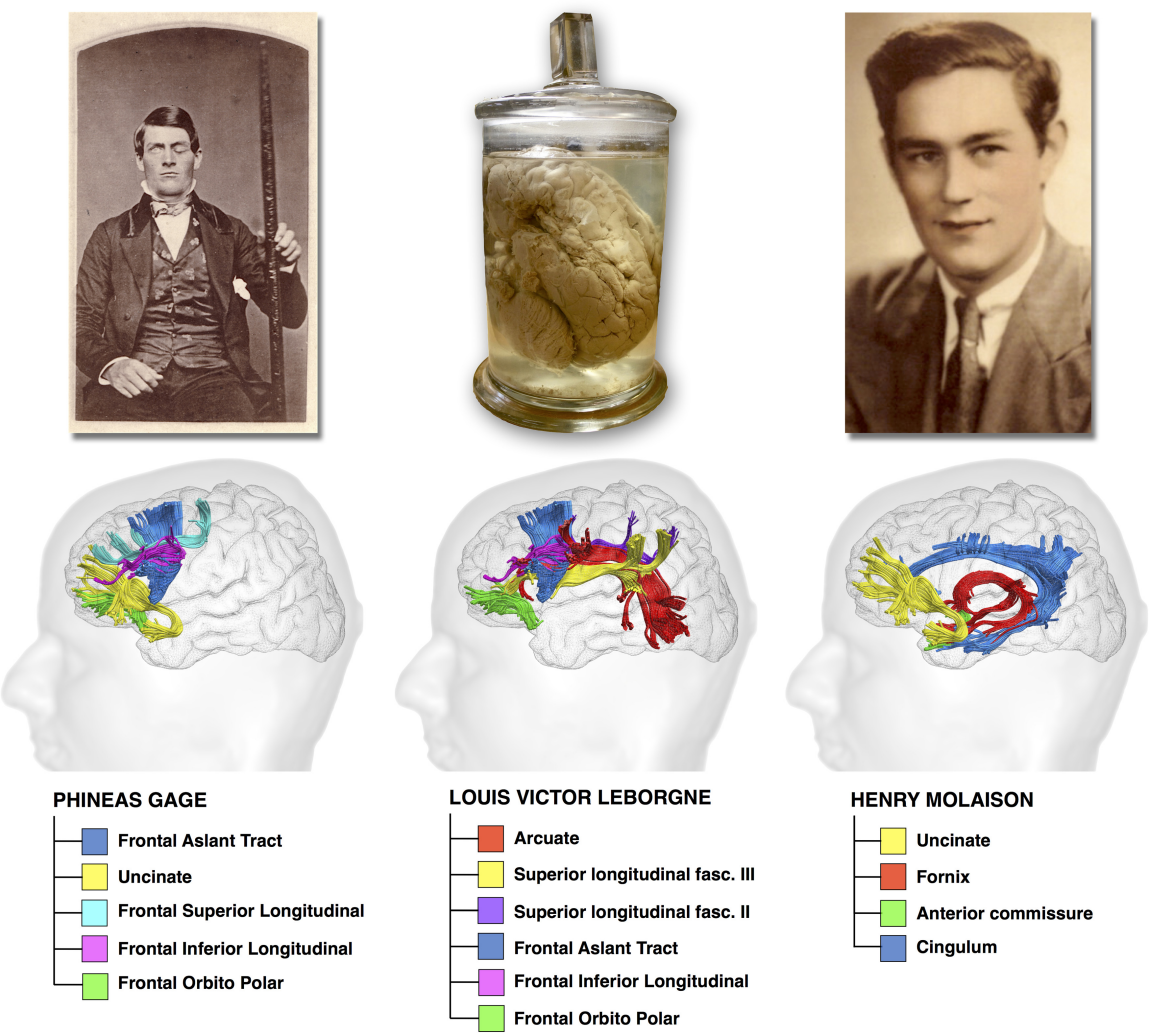
Major tracts that were damaged in Gage (damage affected at least 30% of the tracts' volume, *z*-score = 1.7), in Leborgne (damage affected at least 55% of the tracts' volume, *z*-score = 1.29), and in Molaison (damage affected at least 5% of the tracts' volume, *z*-score = 1.39).

Table 1 lists for each case the percentage of damage to each tract (visually represented in Fig. 4) and the corresponding *z*-score. Figure 5 illustrates the reconstruction of the most damaged tracts in each case.

**Table 1:** Percentage of damage to each tract represented and the corresponding *z*-score for each case

	Gage	Leborgne	Molaison
(1) Fornix	10.9% (0.13)	27.5% (0.21)	6.7% (1.68)
(2) Dorsal cingulum (left)	6.0% (−0.2)	45.4% (0.91)	0.0% (−0.38)
(2) Dorsal cingulum (right)	0.0% (−0.6)	0.9% (−0.83)	0.0% (−0.38)
(3) Ventral cingulum (left)	0.0% (−0.6)	9.4% (−0.5)	6.6% (1.63)
(3) Ventral cingulum (right)	0.0% (−0.6)	0.0% (−0.87)	5.7% (1.39)
(4) Uncinate fasciculus (left)	43.1% (2.29)*	52.6% (1.19)	3.9% (0.82)
(4) Uncinate fasciculus (right)	0.0% (−0.6)	0.0% (−0.87)	17.3% (4.9)***
(5) Fronto striatal (left)	22.8% (0.93)	52.4% (1.19)	0.0% (−0.38)
(5) Fronto striatal (right)	0.0% (−0.6)	0.0% (−0.87)	0.0% (−0.38)
(6) Superior longitudinal fasciculus I (left)	14.0% (0.33)	40.6% (0.73)	0.0% (−0.38)
(6) Superior longitudinal fasciculus I (right)	0.0% (−0.6)	0.0% (−0.87)	0.0% (−0.38)
(7) Superior longitudinal fasciculus II (left)	15.5% (0.44)	64.0% (1.64)	0.0% (−0.38)
(7) Superior longitudinal fasciculus II (right)	0.0% (−0.6)	0.0% (−0.87)	0.0% (−0.38)
(8) Superior longitudinal fasciculus III (left)	19.9% (0.73)	76.6% (2.14)*	0.0% (−0.38)
(8) Superior longitudinal fasciculus III (right)	0.0% (−0.6)	0.0% (−0.87)	0.0% (−0.38)
(9) Cortico-spinal tract (left)	0.0% (−0.6)	46.3% (0.95)	0.0% (−0.38)
(9) Cortico-spinal tract (right)	0.0% (−0.6)	0.0% (−0.87)	0.0% (−0.38)
(10) Anterior thalamic radiations (left)	23.0% (0.94)	52.4% (1.19)	0.0% (−0.38)
(10) Anterior thalamic radiations (right)	0.0% (−0.6)	0.0% (−0.87)	0.0% (−0.38)
(11) Fronto pontine (left)	22.5% (0.91)	50.7% (1.12)	0.0% (−0.38)
(11) Fronto pontine (right)	0.0% (−0.6)	0.0% (−0.87)	0.0% (−0.38)
(12) Arcuate long segment (left)	1.9% (−0.47)	59.5% (1.47)	0.0% (−0.38)
(12) Arcuate long segment (right)	0.0% (−0.6)	0.0% (−0.87)	0.0% (−0.38)
(13) Arcuate posterior segment (left)	0.0% (−0.6)	28.9% (0.26)	0.0% (−0.38)
(13) Arcuate posterior segment (right)	0.0% (−0.6)	0.0% (−0.87)	0.0% (−0.38)
(14) Optic radiations (left)	0.0% (−0.6)	20.5% (−0.06)	0.0% (−0.38)
(14) Optic radiations (right)	0.0% (−0.6)	0.0% (−0.87)	0.0% (−0.38)
(15) Inferior fronto-occipital fasciculus (left)	17.2% (0.55)	39.3% (0.67)	0.0% (−0.38)
(15) Inferior fronto-occipital fasciculus (right)	0.0% (−0.6)	0.0% (−0.87)	0.0% (−0.38)
(16) Inferior longitudinal fasciculus (left)	4.6% (−0.29)	12.5% (−0.38)	1.9% (0.2)
(16) Inferior longitudinal fasciculus (right)	0.0% (−0.6)	0.0% (−0.87)	3.0% (0.55)
(17) Frontal superior longitudinal (left)	47.1% (2.55)**	30.3% (0.32)	0.0% (−0.38)
(17) Frontal superior longitudinal (right)	0.0% (−0.6)	0.0% (−0.87)	0.0% (−0.38)
(18) Frontal inferior longitudinal (left)	50.5% (2.78)**	71.6% (1.94)	0.0% (−0.38)
(18) Frontal inferior longitudinal (right)	0.0% (−0.6)	0.0% (−0.87)	0.0% (−0.38)
(19) Frontal aslant tract (left)	42.9% (2.27)*	55.0% (1.29)	0.0% (−0.38)
(19) Frontal aslant tract (right)	0.0% (−0.6)	0.0% (−0.87)	0.0% (−0.38)
(20) Frontal orbitopolar (left)	34.4% (1.7)	58.6% (1.43)	0.0% (−0.38)
(20) Frontal orbitopolar (right)	0.0% (−0.6)	0.0% (−0.87)	0.0% (−0.38)
(21) Fronto marginal tract (left)	0.0% (−0.6)	9.7% (−0.49)	0.0% (−0.38)
(21) Fronto marginal tract (right)	0.0% (−0.6)	0.0% (−0.87)	0.0% (−0.38)
(22) Anterior commissure	0.0% (−0.6)	24.2% (0.08)	7.0% (1.77)

Note: *P < 0.05; **P < 0.01; ***P < 0.001.

### Phineas Gage

The atlas-based analysis showed that the trajectory of the bar impacted several frontal lobe tracts, including primarily the uncinate fasciculus (43.1%; *z* = 2.287; *P* = 0.022), frontal superior (47.1%; *z* = 2.552; *P* = 0.011) and inferior longitudinal (50.5%; *z* = 2.782; *P* = 0.005) tracts, frontal aslant tract (42.9%; *z* = 2.275; *P* = 0.023), and frontal orbitopolar tract (34.4%; *z* = 1.702; *P* = 0.089) (Table 1) (Fig. 5). Other partially affected tracts included the anterior thalamic projections (23%; *z* = 0.94; *P* = 0.347), fronto-striatal projections (22.8%; *z* = 0.93; *P* = 0.352), and fronto-pontine projections (22.5%; *z* = 0.91; *P* = 0.363). Table 1 contains a complete list of all analyzed tracts.

**Figure 6.**
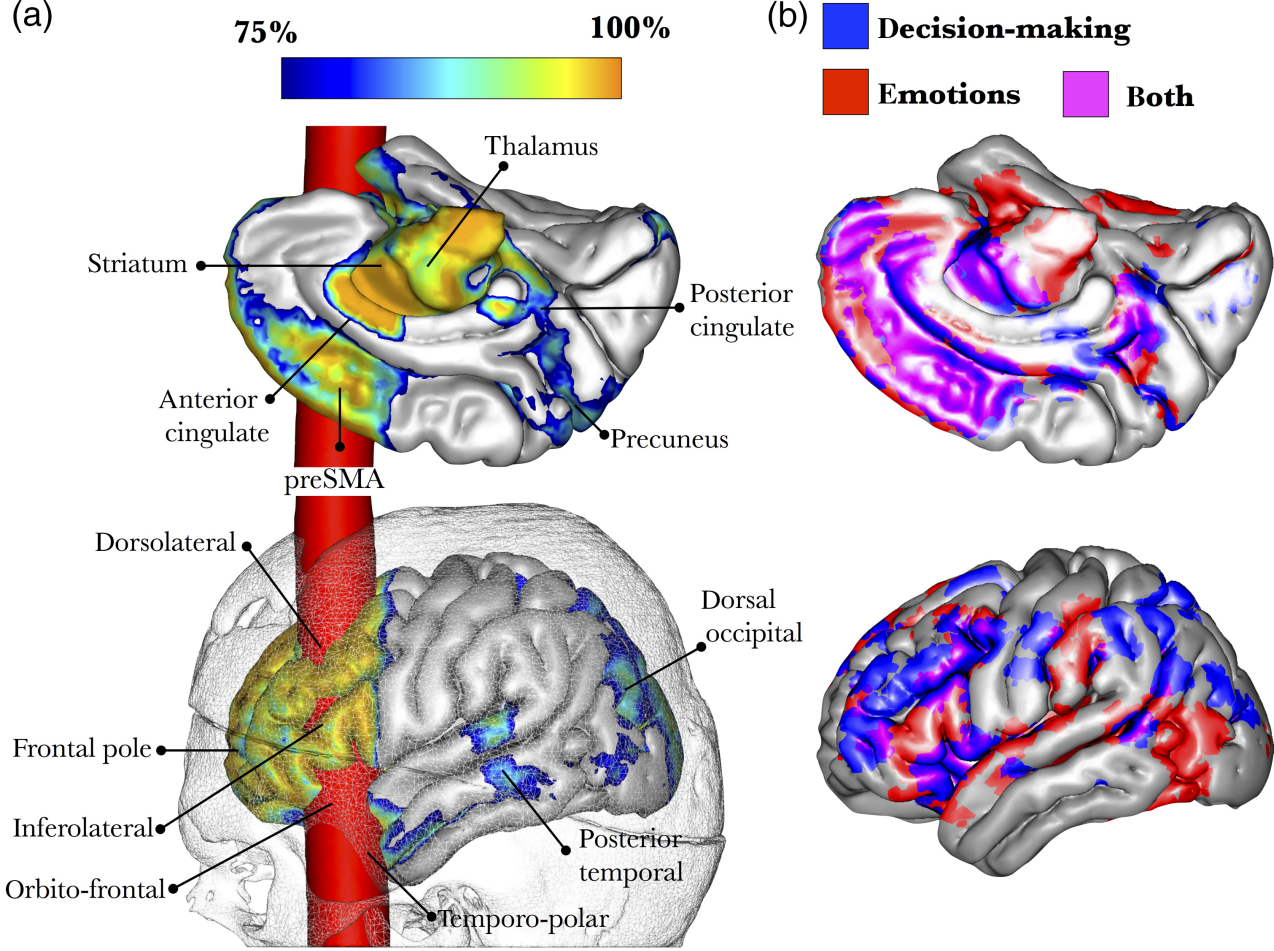
3D reconstruction of Gage's skull co-registered in the MNI space with a brain averaged from 152 subjects. (*a*) The trajectory of the bar through the brain is in red and shows damage to cortical areas and subcortical white matter. A blue-to-orange gradient indicates the probability of disconnection of areas not directly affected by the bar. (*b*) Meta-analysis of functional MRI studies reporting activations related to decision-making tasks (66 studies) and emotions (215 studies).

The lesion-based analysis indicated direct damage to the orbitofrontal cortex, dorsolateral prefrontal cortex, and temporopolar cortex. In addition, the lesion disconnected several areas not directly affected by the tamping iron. These areas include the frontal pole, posterior inferolateral frontal cortex, anterior and posterior cingulate, pre-supplementary motor area, precuneus, posterior temporal, and dorsolateral occipital cortices. Other subcortical structures that were partially disconnected from the frontal lobe were the thalamus, the striatum, and amygdala (Fig. 6a).

The meta-analysis of fMRI studies on decision-making and emotional processing showed an extended network that overlapped the tractography-derived structural networks, except for the inferior parietal regions (Fig. 6b).

### Louis Victor Leborgne

The atlas-based analysis showed that the lesion in Leborgne's brain disconnected several tracts, among which the most significantly damaged were the third branch of the superior longitudinal fasciculus (76.6%; *z* = 2.137; *P* = 0.033), long segment of the arcuate fasciculus (59.5%; *z* = 1.466; *P* = 0.142), frontal inferior longitudinal fasciculus (71.6%; *z* = 1.943; *P* = 0.052), frontal orbitopolar tract (58.6%; *z* = 1.43; *P* = 0.152), and frontal aslant tract (55%; *z* = 1.29; *P* = 0.197) (Table 1) (Fig. 5). Other partially affected tracts included the anterior thalamic radiations (52.4%; *z* = 1.187; *P* = 0.235), cortico-spinal tract (46.3%; *z* = 0.949; *P* = 0.342), frontal striatal tract (52.4%; *z* = 1.19; *P* = 0.234), fronto-pontine tract (50.7%; *z* = 1.122; *P* = 0.261), and the second branch (64%; *z* = 1.643; *P* = 0.100) of the superior longitudinal fasciculus. Table 1 contains a complete list of all analyzed tracts.

**Figure 7.**
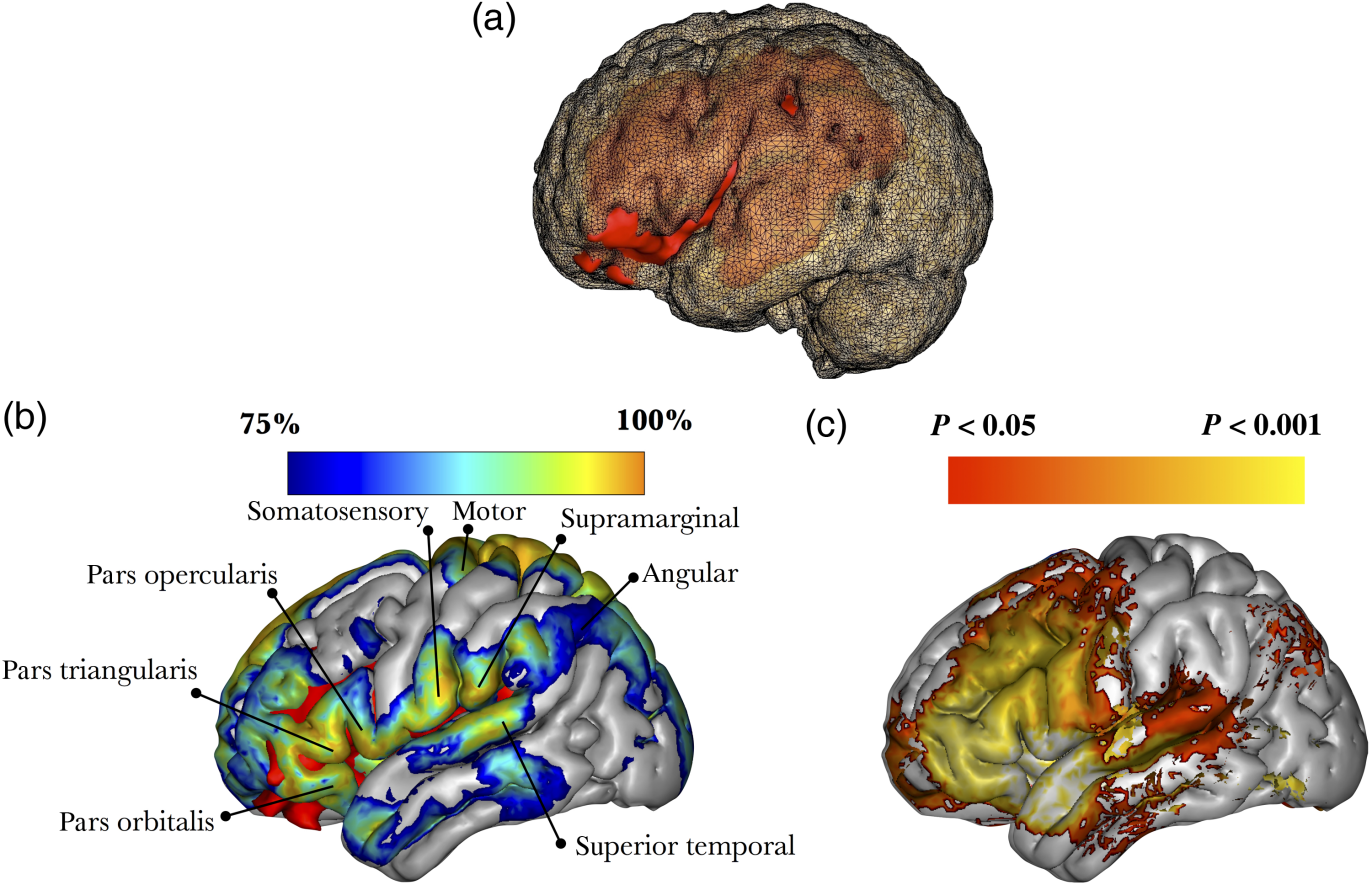
3D reconstruction of Leborgne's brain in MNI space. (*a*) The lesion (red) damaged both cortical structures (posterior inferior frontal cortex) and subcortical white matter (perisylvian pathways). (*b*) 3D reconstruction of the MNI152 template, with a blue-to-orange gradient indicating the probability of disconnection of those areas not directly affected by the lesion; red color indicate damage caused by the lesion. (*c*) Meta-analysis of functional MRI studies reporting activations related to the performance of fluency tasks (75 studies).

The lesion-based analysis indicated that the damage in Leborgne's brain affected an extended network involving not only Broca's territory but also distant regions in the frontal, parietal, and temporal lobes (Fig. 7a).

The meta-analysis of fMRI studies reporting activations for verbal fluency tasks showed a functional network included in the structural network identified by tractography (Fig. 7b). Other regions (e.g., primary motor cortex) affected were not part of the verbal fluency meta-analysis and may account for Leborgne's right hemiplegia.

### Henry Gustave Molaison

Our atlas-based analysis indicated that the tract that was the most significantly damaged was the right uncinate fasciculus (17.3%; *z* = 4.946; *P* < 0.001). Other partially affected tracts included the fornix (6.7%; *z* = 1.678; *P* = 0.093), the anterior commissure (7%; *z* = 1.768; *P* = 0.077), and left (6.6%; *z* = 1.640; *P* = 0.101) and right (5.7% *z* = 1.391; *P* = 0.164) ventral cingulum (Table 1) (Fig. 5).

**Figure 8.**
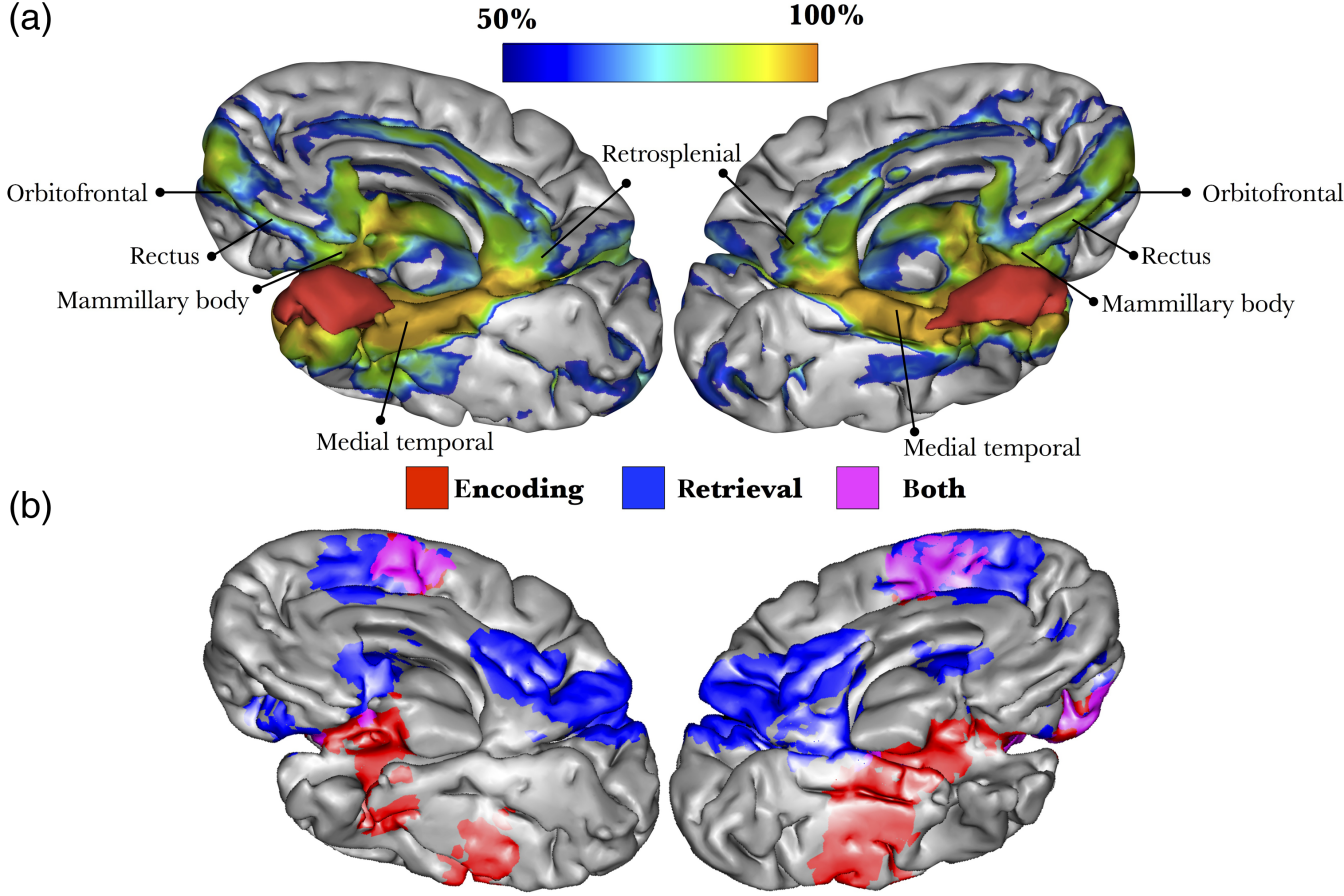
3D reconstruction of Molaison's brain registered in MNI space. (*a*) The damage produced by the surgery (red) affected cortical regions and subcortical white matter. A blue-to-orange gradient indicates the probability of disconnection of areas not directly affected by the excision. (*b*) Meta-analysis of functional MRI studies reporting activations related to encoding and retrieval of declarative memories (Spaniol et al. 2009).

The lesion-based analysis showed that damage in Molaison's brain affected a network of areas, including the medial temporal cortices, retrosplenial cortex, orbitofrontal cortex, and gyrus rectus (Fig. 8a). Other subcortical disconnected regions included the mammillary bodies and septal nuclei.

Using the meta-analysis of fMRI studies carried out by Spaniol et al. (2009), we found that the areas activated during encoding and retrieval of declarative memories overlapped the cortical projections of the tracts compromised in Molaison's brain. These areas included the posterior parahippocampal and retrosplenial cortices, anterior and posterior cingulate gyrus, the dorsomedial prefrontal cortex, precuneus, orbitofrontal cortex, mammillary bodies, and anterior thalamic nuclei (Fig. 8b).

## Discussion

Modern approaches to brain function rely on the ability to map the complexity of brain networks underlying cognition and behavior. In our study, we revisited 3 seminal cases in the history of neurology to better understand the contribution of disconnection to their cognitive and behavioral syndromes. Our analysis showed that in all 3 cases, the damage compromised both short- and long-range connections, suggesting that disconnection mechanisms occurred beyond the lesion site. Below we discuss these findings in greater detail for each patient.

### Phineas Gage

Previous computerized reconstructions of Gage's skull indicated damage localized to frontal to cortical regions (orbitofrontal and dorsolateral prefrontal cortex) (Damasio et al. 1994; Ratiu and Talos 2004a,b) and underlying white matter (Van Horn et al. 2012). In our study, we identified disconnections in 3 major white matter networks: the uncinate fasciculus linking the orbitofrontal to the anterior temporal lobe; frontal intralobar networks (connections between frontal regions); and the fronto-striatal-thalamo-frontal network.

The *uncinate fasciculus* connects the anterior temporal regions (entorhinal cortex, amygdala, temporopolar cortex) with medial and lateral orbitofrontal cortex (Crosby et al. 1962). Temporal regions connected by the uncinate fasciculus are involved in episodic and semantic memory and emotion (Von Der Heide et al. 2013; Murray et al. 2014). The orbitofrontal regions are associated with response inhibition, mood regulation, and reward (Berlin et al. 2004; Kramer et al. 2013; Kumfor et al. 2013). Lesions to the anterior temporal and orbitofrontal regions or their connections often cause mood and behavioral symptoms. In traumatic brain injury, for example, patients with lesions to this anterior orbitofrontal-temporal network show socially inappropriate and disinhibited behavior, impulsivity, compulsive eating, reduced control of emotional response, reduced empathy, rigidity, and perseveration (Zappala et al. 2012; Dal Monte et al. 2014). Patients with anterior temporal lobe epilepsy may also manifest delusions and hallucinations. Damage to the uncinate fasciculus and its cortical projections has been reported in children with conduct disorder (Sarkar et al. 2013) and adults with psychopathy (Craig et al. 2009). Hence, the disconnection of the left uncinate fasciculus in Gage may account for some of the behavioral manifestations reported by Harlow (1868). The uncinate fasciculus has been also associated with semantic deficits in patients with neurodegenerative disorders (Catani, Dell'acqua et al. 2013). In the original accounts, Harlow did not report whether Gage showed impairment in naming or semantic knowledge. One may speculate that damage to the uncinate fasciculus was limited to the medial “limbic” portion of the uncinate fasciculus, leaving the most lateral projections to Broca's area intact.

The frontal intralobar networks include 3 sets of connections between different regions of the frontal lobe: the fronto-orbitopolar tract, frontal aslant tract, and frontal superior and inferior longitudinal tracts (Catani, Dell'acqua, Vergani et al. 2012; Thiebaut de Schotten et al. 2012).

The frontal orbitopolar tract represents a transmodal network for binding memories and emotions with olfactory, taste, visual, and auditory inputs. Multisensory association and limbic integration are important to guide complex cognitive and behavioral functions, such as reward behavior associated with sensory and abstract reinforcers (e.g., monetary gain and loss) (Kringelbach 2005) or response inhibition (e.g., go-no-go tasks) (Iversen and Mishkin 1970).

The frontal aslant tract connects Broca's territory with medial frontal areas (including the pre-supplementary motor area and cingulate cortex) (Lawes et al. 2008; Oishi et al. 2008; Ford et al. 2010; Guevara et al. 2011). In patients with traumatic brain injury, damage to the frontal aslant tract is correlated with impaired response inhibition (Bonnelle et al. 2012). Interestingly, the strength of activation of the inferior frontal gyrus and the pre-supplementary motor area during fMRI-based response inhibition tasks has been associated with recurrent antisocial behavior (Aharoni et al. 2013). Other deficits include speech initiation problems, from which patients often recover due to the bilateral distribution of this tract (see discussion for Leborgne).

The frontal superior and inferior longitudinal tracts connect regions of the frontal lobe involved in decision-making at different levels, from a low-processing level in the posterior frontal regions to a high-processing level in more anterior frontal regions (Badre and D'Esposito 2007; Badre 2008; Christoff et al. 2009). Overall, these longitudinal tracts permit the anatomical binding necessary for complex cognitive control (Koechlin et al. 1999, 2003; Koechlin and Summerfield 2007). While more posterior frontal regions appeared intact in Gage's brain, damage to the connections between posterior and anterior frontal regions could explain his deficits in high-level cognitive control.

The fronto-striatal and thalamo-frontal networks form parallel cortico-subcortical loops for motor, associative, and limbic processing (Alexander et al. 1986; Lehericy et al. 2004; Schmahmann and Pandya 2008).

The associative circuit subsumes the dorsolateral prefrontal cortex, dorsal caudate nucleus, internal pallidum, and ventral anterior thalamic nuclei. Lesions to the associative circuit impair attention, working memory, strategy formation, and cognitive flexibility (Stuss and Benson 1984). The limbic circuit incorporates the medial and orbitofrontal cortices, ventral striatum (i.e., nucleus accumbens), external and internal pallidum, and mediodorsal thalamic nucleus. Functions of the limbic loop overlap with those of the fronto-orbitopolar tract described above. Gage had significant damage to this loop, although the exact extent of the lesion is difficult to quantify using our indirect approach.

Overall, our analysis uncovered extensive frontal lobe damage in Gage's brain. This abnormality extended beyond the orbitofrontal and dorsolateral cortices, which were directly damaged by the bar. The atlas-based approach identified several tracts affected by the lesion, and the lesion-based approach showed that the dysfunction impacted an extended network of areas that are commonly activated during the performance of decision-making, emotion processing, and reward tasks.

### Louis Victor Leborgne

Soon after Broca's (1861a,b) publication, the concept of a center for spoken language was harshly criticized by Pierre Marie (1906), Henry Head (1926), and many others. Their dissent was based on empirical evidence of the existence of patients with non-fluent aphasia without damage to Broca's area. Broca was also criticized for not performing dissections of the whole brain but limiting his investigation to the cortical surface. Indeed, when computerized tomography (CT) and magnetic resonance imaging (MRI) scans of Leborgne's brain were published, it was evident that the lesion extended well beyond the inferior frontal gyrus to include large regions of the underlying white matter (Castaigne et al. 1980; Signoret et al. 1984; Cabanis et al. 1994; Dronkers et al. 2007). Our study confirmed that the extensive lesion in Leborgne's brain affected almost all dorsolateral tracts of the left hemisphere, including the arcuate fasciculus and frontal aslant tract, both of which support language.

The long segment of the arcuate fasciculus connects Wernicke's with Broca's region, whereas the anterior segment of the arcuate fasciculus (or third branch of the superior longitudinal fasciculus) connects Broca's to Geschwind's territory (in the inferior parietal lobule) (Catani et al. 2005). In addition, the frontal aslant tract connects Broca's to the pre-supplementary area. These 3 tracts constitute a complex network dedicated to speech production (Roelofs 2014). In Leborgne, whose only verbal output was limited to a few words, the lesion to these 3 tracts explains his poor verbal fluency. Further, our analysis suggested that Leborgne's pathology extended to tracts that are not part of the language system. The left cortico-spinal tract, for example, was damaged at different levels (corona radiata, internal capsule), which accounts for his right hemiplegia.

It is difficult to say whether damage to other tracts, such as the uncinate fasciculus, frontal inferior longitudinal fasciculus, fronto-orbitopolar tracts, and superior longitudinal fasciculus had an impact on Leborgne's behavior. Broca did not report any other significant impairment and noted that Leborgne had normal intelligence. There is no mention, for example, of limb apraxia, which is usually associated with left hemisphere damage to the superior longitudinal fasciculus. Similarly, damage to the frontal orbitopolar tract may have caused behavioral problems that were not reported in the case notes. It is also true that Broca's knowledge of the patient was minimal and limited to a surgical consultation for the gangrenous leg. In the absence of more detailed clinical notes, speculation about possible symptoms caused by tracts not directly involved in language is risky.

### Henry Molaison

Molaison's groundbreaking case established that bilateral medial temporal lobe lesions cause severe amnesia (Scoville and Milner 1957). MRI studies carried out in 1992 and 1993 showed that the resection included the medial temporal polar cortex, most of the amygdaloid complex, and all of the entorhinal cortex (Corkin et al. 1997). Also removed were the anterior ∼2 cm of the dentate gyrus, hippocampus, and subicular complex, and the rostral portions of perirhinal and parahippocampal cortices.

Molaison's memory impairment was more severe than that of amnesic patients with selective hippocampal lesions (Zola-Morgan et al. 1994), suggesting that damage to his entorhinal, perirhinal, and parahippocampal cortices exacerbated the deficit. Lesion studies in monkeys and fMRI studies in humans provide abundant evidence that these areas are recruited during the performance of declarative memory tasks, yet the contribution to memory processes from the preserved caudal portion of Molaison's perirhinal and parahippocampal cortices could not support normal memory performance (Corkin et al. 1997; von Allmen et al. 2014).

Our findings are consistent with the view that medial temporal lobe structures are a part of an extended network of cortical and subcortical structures that support memory consolidation, storage, and retrieval (Scoville and Milner 1957; Warrington 1985; Markowitsch 2000; Gaffan et al. 2001, 2002; Moulin et al. 2013; Annese et al. 2014). The fornix is a medial structure composed of commissural and projection fibers. The majority of the fibers of the fornix connect the hippocampus with the mammillary bodies, the anterior thalamic nuclei, and the hypothalamus; the fornix also has a small commissural component known as the hippocampal commissure (Crosby et al. 1962; Aggleton 2008; Nieuwenhuys et al. 2008). Damage to fibers of the fornix in Molaison may have contributed to his anterograde memory deficits, but the profound memory impairment cannot be explained entirely by the fornix disconnection. Aggleton (2008) showed that the anterograde amnesia observed with disconnection of the fornix without damage to the medial temporal lobe is not as severe as that seen in patients with bilateral hippocampal damage. The milder impairment with fornix lesions may stem from the fact that information from the hippocampus can travel to other structures of the limbic system through alternative pathways, such as the ventral cingulum (Vann and Albasser 2011) and uncinate fasciculus (Metzler-Baddeley et al. 2012). Both pathways were affected in Molaison. The damage to the connections to the orbitofrontal cortex (via the uncinate fasciculus) and precuneus (via the posterior cingulum) is noteworthy, because these areas are affected in neurodegenerative disorders involving memory such as mild cognitive impairment and early Alzheimer disease (Acosta-Cabronero et al. 2012).

For the most part, Molaison's preoperative semantic knowledge was intact and did not deteriorate from 1953 (preoperatively) to 2000 (Kensinger et al. 2001; Steinvorth et al. 2005). He did, however, show deficits on category and letter fluency tasks and tests of definitions (Kensinger et al. 2001; Schmolck et al. 2002). Several factors may account for his poor performance: low socioeconomic background, substandard education, slow response times, and damage to temporal neocortex. Semantic memory relies on a large, distributed cortical network that includes areas in the inferior frontal gyrus and the temporal pole bilaterally (Martin and Chao 2001; Hoffman et al. 2014). The latter 2 regions are interconnected through the uncinate fasciculus and the anterior commissure, which were damaged in Molaison's brain. Bilateral abnormalities of the uncinate fasciculus have been associated with semantic deficits in neurodegenerative disorders (Mummery et al. 1999; Compston 2011a,b; Catani, Mesulam et al. 2013), and the disconnection of these tracts may have contributed to his shortcomings on certain semantic tasks.

Axonal tracing studies in animals show that the anterior fibers of the anterior commissure also project to olfactory regions (e.g., olfactory bulb, anterior perforated substance, etc.) (Crosby et al. 1962). These anterior olfactory-linked fibers seem to be present in humans (Kiernan 1998; Di Virgilio et al. 1999), and the damage to these tracts that we have identified in H.M. may have contributed to his deficit in odor quality discrimination and recognition (Eichenbaum et al. 1983).

MRI scans and more recent postmortem examination of Molaison's brain confirmed that the medial temporal stem was partially excised and white matter anatomy appeared significantly altered (Augustinack et al. 2014). It is likely that the surgery damaged other small white matter pathways in addition to those detected by our tractography analysis. In particular, it is highly probable that damage included the perforant fibers between the hippocampus and the entorhinal cortex, which are important in memory processes (Witter et al. 2000), or the fibers of the stria terminalis linking amygdala to the hypothalamus and involved in the regulation of adrenergic response to acute stress. In addition, the mammillary nuclei, which receive projections from the hippocampi through the fornix, were recently reported as shrunken in a postmortem study of Molaison's brain (Annese et al. 2014).

### General Discussion and Limitations

This study demonstrated the validity of applying an atlas-based approach to reappraise the effects of disconnection in 3 historic patients for whom data on lesion location and clinical deficits were available. Still, a note of caution is in order because each step used in our analysis presented challenges that could have generated artifactual results.

The absence of the brain, as in the case of Phineas Gage, and the deformation of Leborgne's brain due to preservation in a jar for more than 150 years posed difficulties when we tried to map the real extent of the lesion (Clark et al. 2003). Further, Leborgne's case is problematic as 21 years elapsed between the onset of his language deficits and his death. This is a limitation as Leborgne's brain lesion may have become more extensive or additional lesions not related to his language deficits may have occurred in the subsequent years of his adult life. In addition, delineating a precise margin between pathological and normal tissue is particularly challenging (Seghier et al. 2008). Once the borders of the lesion were reconstructed, mapping the lesion onto the tract was often prone to biases related to misregistration, incorrect estimation of the size of the tracts, and interindividual variability in white matter anatomy (Crinion et al. 2007; Jones et al. 2013).

The lack of diffusion weighted imaging images for the 3 cases led us to gather indirect anatomical information from a data set of 129 normal men and women aged 18–79 years. To obtain an average representation of the anatomy of each tract from the whole sample, several steps were necessary, including registration, overlapping, and thresholding. Thus, while the atlas generated after these steps provided an overall estimation of the tracts' anatomy, it may not precisely match the exact individual anatomy of the 3 patients.

Further, the diffusion-weighted imaging that we have used for this analysis is based on a population of 18- to 79-year-old healthy participants and is not age or sex matched for each historic patient. Age-related changes in volume and diffusion indices of white matter pathways have been reported in previous studies (Stadlbauer et al. 2008; Rojkova et al. 2015). Similarly, sex-related differences have been reported for the right arcuate fasciculus (Catani et al. 2007). These differences can lead to under- or overestimation of the exact extension of the lesion. For this reason, we have included in our interpretation those tracts that showed a trend towards significant disconnection.

It should also be noted that tract estimation was based on tractography, which has many flaws. Even with more advanced diffusion methods, like spherical deconvolution, artifacts can occur due to partial volume effects and difficulty in reconstructing complex anatomical configurations (e.g., crossing, kissing, and fanning) and low spatial resolution (Dell'Acqua and Catani 2012; Kristo et al. 2013). These drawbacks could lead to under- or overestimation of the real anatomy of the tracts with consequences for the atlas-based approach. For example, if the real extent of a tract is underestimated, the atlas-based analysis may incorrectly indicate a relative sparing of the underestimated tract. White matter pathways also show a descending gradient of intersubject variability going from the stem portion (>90% of the population studied) of the white matter pathways to the most peripheral zones (<50% of the population studied; Thiebaut de Schotten, ffytche et al. 2011). In our analysis, we chose probabilities above 50% to consider only the almost invariable anatomical core of each single tract and not its periphery (Thiebaut de Schotten, ffytche et al. 2011).

Another limitation is the lack of a precise quantification of the severity of the disconnection. We used as a surrogate measure of tract damage the proportion of the tract that was intersected by the lesion. While this measure can provide an approximate estimate of the overall involvement of the tract, it does not indicate whether the lesion affected critical fibers. For example, a small lesion located in the internal capsule could lead to a greater functional impairment of the functions related to the cortico-spinal tract than a larger lesion in the corona radiata.

Unfortunately, the historic descriptions of the behavioral symptoms manifested by Gage and Leborgne remain incomplete. Indeed, these observations occurred at the dawn of behavioral neurology, and it is very difficult to back-trace definitive clinico-anatomical conclusions. Further, many connections of the human brain are unknown, and some tracts were not included in the analysis (most of the u-shaped fibers). Clinico-anatomical correlations were particularly difficult in Gage and Leborgne due to the lack of detailed information on the clinical manifestations and evolution of their disorders (e.g., extent of recovery). Lesions and symptoms change over time and could lead to modifications of the link between brain and behavior (Kolb and Gibb 2014; Papagno and Vallar 2014). For example, while Gage recovered his behavioral functions to some extent and could even hold a job, he eventually developed epilepsy and other symptoms (Macmillan 2002). Recovery might be related to “reserve” networks, such as preserved structures on the right hemisphere (Geschwind 1965a,b; Forkel et al. 2014), which might account for symptom improvement (Duffau 2014).

Finally, mapping symptoms onto single tracts is subject to some criticisms. Cortical lesions by definition destroy the white matter tracts associated with them (Geschwind 1965a,b) and in pure white matter lesions the cortex is not affected. This suggests that a network dysfunction is the common denominator for all brain disorders, and a tract-based nomenclature should be preferred to a cortical localizationism. However, syndromes certainly result from a dysfunction of an extended network of cortical and subcortical areas connected by several tracts (Damasio and Damasio 1989). Hence, mapping the disconnection in patients should not lead to an underestimation of the role of the cortex. Indeed, our lesion-based analysis (Figs 6a, 7b, and 8a) revealed cortical regions that were directly or indirectly affected by the disconnection.

## Conclusions

Today as 50 years ago, the clinico-anatomical correlation method remains pivotal in our understanding of the complex relations between brain and behavior (Mah et al. 2014). The disconnection paradigm, as envisaged by Geschwind in his landmark paper and revitalized today by the availability of methods for mapping connections in the living human brain, is key to a comprehensive approach to probing its complexity. Our findings suggest that social behavior, language, and memory depend on the coordinated activity of different regions rather than single areas in the frontal or temporal lobes. While the exact contribution of cortical and disconnection mechanisms remains to be defined with more precision (Critchley 1953; Mesulam 1981, 2005; Damasio 1989), our findings suggest that insights from famous cases that greatly contributed to the advance of neurological knowledge should not be considered to be set in stone.

## Supplementary material

Supplementary material can be found at: http://www.cercor.oxfordjournals.org/

## Funding

This study represents independent research in part funded by the National Institute for Health Research (NIHR) Biomedical Research Centre at South London and Maudsley NHS Foundation Trust and King's College London. The views expressed are those of the author and not necessarily those of the NHS, the NIHR or the Department of Health. Funding to pay the Open Access publication charges for this article was provided by the Welcome Trust.

## Supplementary Material

Supplementary Data
