# Supplementary material for: From Phineas Gage and Monsieur Leborgne to H.M.: Revisiting Disconnection Syndromes
Source: Cereb Cortex. 2015 Aug 12;25(12):4812–27. doi: 10.1093/cercor/bhv173 (PMC4635921; doi:10.1093/cercor/bhv173)
Supplement: Supplementary Data [file supp_25_12_4812__index.html]

From Phineas Gage and Monsieur Leborgne to H.M.: Revisiting Disconnection Syndromes — From Phineas Gage and Monsieur Leborgne to H.M.: Revisiting Disconnection Syndromes — From Phineas Gage and Monsieur Leborgne to H.M.: Revisiting Disconnection Syndromes — Supplementary Data 

# From Phineas Gage and Monsieur Leborgne to H.M.: Revisiting Disconnection Syndromes

## Supplementary Data

Supplementary Data

- Supplementary References 1 - docx file
- Supplementary References 2 - docx file
- Supplementary References 3 - docx file
