# Supplementary material for: From Phineas Gage and Monsieur Leborgne to H.M.: Revisiting Disconnection Syndromes
Source: Cereb Cortex. 2015 Aug 12;25(12):4812–27. doi: 10.1093/cercor/bhv173 (PMC4635921; doi:10.1093/cercor/bhv173)
Supplement: Supplementary Data [file supp_bhv173_bhv173supp_References1.docx]

**Supplementary References 1**: List of the 66 journal articles included in the decision-making meta-analysis.

Baas D, Aleman A, Vink M, Ramsey NF, de Haan EH, Kahn RS. Evidence of altered cortical and amygdala activation during social decision-making in schizophrenia. NeuroImage. 2008;40(2):719-27.

Bolla KI, Eldreth DA, Matochik JA, Cadet JL. Neural substrates of faulty decision-making in abstinent marijuana users. NeuroImage. 2005;26(2):480-92.

Canessa N, Motterlini M, Alemanno F, Perani D, Cappa SF. Learning from other people's experience: a neuroimaging study of decisional interactive-learning. NeuroImage. 2011;55(1):353-62.

Chang LJ, Sanfey AG. Great expectations: neural computations underlying the use of social norms in decision-making. Social cognitive and affective neuroscience. 2013;8(3):277-84.

Christakou A, Brammer M, Rubia K. Maturation of limbic corticostriatal activation and connectivity associated with developmental changes in temporal discounting. NeuroImage. 2011;54(2):1344-54.

Cohen MX. Individual differences and the neural representations of reward expectation and reward prediction error. Social cognitive and affective neuroscience. 2007;2(1):20-30.

Cohen MX, Heller AS, Ranganath C. Functional connectivity with anterior cingulate and orbitofrontal cortices during decision-making. Brain research Cognitive brain research. 2005;23(1):61-70.

Crowley TJ, Dalwani MS, Mikulich-Gilbertson SK, Du YP, Lejuez CW, Raymond KM, et al. Risky decisions and their consequences: neural processing by boys with Antisocial Substance Disorder. PloS one. 2010;5(9):e12835.

Cunningham WA, Johnsen IR, Waggoner AS. Orbitofrontal cortex provides cross-modal valuation of self-generated stimuli. Social cognitive and affective neuroscience. 2011;6(3):286-93.

Downar J, Bhatt M, Montague PR. Neural correlates of effective learning in experienced medical decision-makers. PloS one. 2011;6(11):e27768.

Engelmann JB, Tamir D. Individual differences in risk preference predict neural responses during financial decision-making. Brain research. 2009;1290:28-51.

Eshel N, Nelson EE, Blair RJ, Pine DS, Ernst M. Neural substrates of choice selection in adults and adolescents: development of the ventrolateral prefrontal and anterior cingulate cortices. Neuropsychologia. 2007;45(6):1270-9.

Feinstein JS, Stein MB, Paulus MP. Anterior insula reactivity during certain decisions is associated with neuroticism. Social cognitive and affective neuroscience. 2006;1(2):136-42.

Fishbein DH, Eldreth DL, Hyde C, Matochik JA, London ED, Contoreggi C, et al. Risky decision making and the anterior cingulate cortex in abstinent drug abusers and nonusers. Brain research Cognitive brain research. 2005;23(1):119-36.

Fleck MS, Daselaar SM, Dobbins IG, Cabeza R. Role of prefrontal and anterior cingulate regions in decision-making processes shared by memory and nonmemory tasks. Cerebral cortex. 2006;16(11):1623-30.

Frangou S, Kington J, Raymont V, Shergill SS. Examining ventral and dorsal prefrontal function in bipolar disorder: a functional magnetic resonance imaging study. European psychiatry : the journal of the Association of European Psychiatrists. 2008;23(4):300-8.

Grossman M, Eslinger PJ, Troiani V, Anderson C, Avants B, Gee JC, et al. The role of ventral medial prefrontal cortex in social decisions: converging evidence from fMRI and frontotemporal lobar degeneration. Neuropsychologia. 2010;48(12):3505-12.

Guroglu B, van den Bos W, Rombouts SA, Crone EA. Unfair? It depends: neural correlates of fairness in social context. Social cognitive and affective neuroscience. 2010;5(4):414-23.

Guroglu B, van den Bos W, van Dijk E, Rombouts SA, Crone EA. Dissociable brain networks involved in development of fairness considerations: understanding intentionality behind unfairness. NeuroImage. 2011;57(2):634-41.

Han SD, Boyle PA, Arfanakis K, Fleischman DA, Yu L, Edmonds EC, et al. Neural intrinsic connectivity networks associated with risk aversion in old age. Behavioural brain research. 2012;227(1):233-40.

Hartstra E, Oldenburg JF, Van Leijenhorst L, Rombouts SA, Crone EA. Brain regions involved in the learning and application of reward rules in a two-deck gambling task. Neuropsychologia. 2010;48(5):1438-46.

Hollmann M, Rieger JW, Baecke S, Lutzkendorf R, Muller C, Adolf D, et al. Predicting decisions in human social interactions using real-time fMRI and pattern classification. PloS one. 2011;6(10):e25304.

Hon N, Epstein RA, Owen AM, Duncan J. Frontoparietal activity with minimal decision and control. The Journal of neuroscience : the official journal of the Society for Neuroscience. 2006;26(38):9805-9.

Ibarretxe-Bilbao N, Junque C, Tolosa E, Marti MJ, Valldeoriola F, Bargallo N, et al. Neuroanatomical correlates of impaired decision-making and facial emotion recognition in early Parkinson's disease. The European journal of neuroscience. 2009;30(6):1162-71.

Ivanoff J, Branning P, Marois R. fMRI evidence for a dual process account of the speed-accuracy tradeoff in decision-making. PloS one. 2008;3(7):e2635.

Jarcho JM, Berkman ET, Lieberman MD. The neural basis of rationalization: cognitive dissonance reduction during decision-making. Social cognitive and affective neuroscience. 2011;6(4):460-7.

Jollant F, Lawrence NS, Olie E, O'Daly O, Malafosse A, Courtet P, et al. Decreased activation of lateral orbitofrontal cortex during risky choices under uncertainty is associated with disadvantageous decision-making and suicidal behavior. NeuroImage. 2010;51(3):1275-81.

Jones CL, Minati L, Harrison NA, Ward J, Critchley HD. Under pressure: response urgency modulates striatal and insula activity during decision-making under risk. PloS one. 2011;6(6):e20942.

Keri S, Decety J, Roland PE, Gulyas B. Feature uncertainty activates anterior cingulate cortex. Human brain mapping. 2004;21(1):26-33.

Kotani Y, Ohgami Y, Kuramoto Y, Tsukamoto T, Inoue Y, Aihara Y. The role of the right anterior insular cortex in the right hemisphere preponderance of stimulus-preceding negativity (SPN): an fMRI study. Neuroscience letters. 2009;450(2):75-9.

Kovacs G, Cziraki C, Greenlee MW. Neural correlates of stimulus-invariant decisions about motion in depth. NeuroImage. 2010;51(1):329-35.

Krain AL, Gotimer K, Hefton S, Ernst M, Castellanos FX, Pine DS, et al. A functional magnetic resonance imaging investigation of uncertainty in adolescents with anxiety disorders. Biological psychiatry. 2008;63(6):563-8.

Krain AL, Wilson AM, Arbuckle R, Castellanos FX, Milham MP. Distinct neural mechanisms of risk and ambiguity: a meta-analysis of decision-making. NeuroImage. 2006;32(1):477-84.

Kruschwitz JD, Simmons AN, Flagan T, Paulus MP. Nothing to lose: processing blindness to potential losses drives thrill and adventure seekers. NeuroImage. 2012;59(3):2850-9.

Lawrence NS, Jollant F, O'Daly O, Zelaya F, Phillips ML. Distinct roles of prefrontal cortical subregions in the Iowa Gambling Task. Cerebral cortex. 2009;19(5):1134-43.

Leland DS, Arce E, Feinstein JS, Paulus MP. Young adult stimulant users' increased striatal activation during uncertainty is related to impulsivity. NeuroImage. 2006;33(2):725-31.

Li J, McClure SM, King-Casas B, Montague PR. Policy adjustment in a dynamic economic game. PloS one. 2006;1:e103.

Litt A, Plassmann H, Shiv B, Rangel A. Dissociating valuation and saliency signals during decision-making. Cerebral cortex. 2011;21(1):95-102.

Lopez-Larson MP, Bogorodzki P, Rogowska J, McGlade E, King JB, Terry J, et al. Altered prefrontal and insular cortical thickness in adolescent marijuana users. Behavioural brain research. 2011;220(1):164-72.

Luo S, Ainslie G, Giragosian L, Monterosso JR. Behavioral and neural evidence of incentive bias for immediate rewards relative to preference-matched delayed rewards. The Journal of neuroscience : the official journal of the Society for Neuroscience. 2009;29(47):14820-7.

Luo S, Ainslie G, Giragosian L, Monterosso JR. Striatal hyposensitivity to delayed rewards among cigarette smokers. Drug and alcohol dependence. 2011;116(1-3):18-23.

Luo S, Ainslie G, Pollini D, Giragosian L, Monterosso JR. Moderators of the association between brain activation and farsighted choice. NeuroImage. 2012;59(2):1469-77.

Marsh AA, Blair KS, Vythilingam M, Busis S, Blair RJ. Response options and expectations of reward in decision-making: the differential roles of dorsal and rostral anterior cingulate cortex. NeuroImage. 2007;35(2):979-88.

Marsh AA, Finger EC, Fowler KA, Jurkowitz IT, Schechter JC, Yu HH, et al. Reduced amygdala-orbitofrontal connectivity during moral judgments in youths with disruptive behavior disorders and psychopathic traits. Psychiatry research. 2011;194(3):279-86.

Monterosso JR, Ainslie G, Xu J, Cordova X, Domier CP, London ED. Frontoparietal cortical activity of methamphetamine-dependent and comparison subjects performing a delay discounting task. Human brain mapping. 2007;28(5):383-93.

Northoff G, Grimm S, Boeker H, Schmidt C, Bermpohl F, Heinzel A, et al. Affective judgment and beneficial decision making: ventromedial prefrontal activity correlates with performance in the Iowa Gambling Task. Human brain mapping. 2006;27(7):572-87.

Novais-Santos S, Gee J, Shah M, Troiani V, Work M, Grossman M. Resolving sentence ambiguity with planning and working memory resources: Evidence from fMRI. NeuroImage. 2007;37(1):361-78.

Paulus MP, Feinstein JS, Leland D, Simmons AN. Superior temporal gyrus and insula provide response and outcome-dependent information during assessment and action selection in a decision-making situation. NeuroImage. 2005;25(2):607-15.

Paulus MP, Frank LR. Anterior cingulate activity modulates nonlinear decision weight function of uncertain prospects. NeuroImage. 2006;30(2):668-77.

Paulus MP, Lovero KL, Wittmann M, Leland DS. Reduced behavioral and neural activation in stimulant users to different error rates during decision making. Biological psychiatry. 2008;63(11):1054-60.

Rao H, Korczykowski M, Pluta J, Hoang A, Detre JA. Neural correlates of voluntary and involuntary risk taking in the human brain: an fMRI Study of the Balloon Analog Risk Task (BART). NeuroImage. 2008;42(2):902-10.

Roy AK, Gotimer K, Kelly AM, Castellanos FX, Milham MP, Ernst M. Uncovering putative neural markers of risk avoidance. Neuropsychologia. 2011;49(5):937-44.

Sato M, Baciu M, Loevenbruck H, Schwartz JL, Cathiard MA, Segebarth C, et al. Multistable representation of speech forms: a functional MRI study of verbal transformations. NeuroImage. 2004;23(3):1143-51.

Schleim S, Spranger TM, Erk S, Walter H. From moral to legal judgment: the influence of normative context in lawyers and other academics. Social cognitive and affective neuroscience. 2011;6(1):48-57.

Shad MU, Bidesi AS, Chen LA, Thomas BP, Ernst M, Rao U. Neurobiology of decision-making in adolescents. Behavioural brain research. 2011;217(1):67-76.

Sharp DJ, Awad M, Warren JE, Wise RJ, Vigliocco G, Scott SK. The neural response to changing semantic and perceptual complexity during language processing. Human brain mapping. 2010;31(3):365-77.

Sharp DJ, Scott SK, Mehta MA, Wise RJ. The neural correlates of declining performance with age: evidence for age-related changes in cognitive control. Cerebral cortex. 2006;16(12):1739-49.

Simmons A, Miller D, Feinstein JS, Goldberg TE, Paulus MP. Left inferior prefrontal cortex activation during a semantic decision-making task predicts the degree of semantic organization. NeuroImage. 2005;28(1):30-8.

Sip KE, Lynge M, Wallentin M, McGregor WB, Frith CD, Roepstorff A. The production and detection of deception in an interactive game. Neuropsychologia. 2010;48(12):3619-26.

Smith BW, Mitchell DG, Hardin MG, Jazbec S, Fridberg D, Blair RJ, et al. Neural substrates of reward magnitude, probability, and risk during a wheel of fortune decision-making task. NeuroImage. 2009;44(2):600-9.

Sommer M, Rothmayr C, Dohnel K, Meinhardt J, Schwerdtner J, Sodian B, et al. How should I decide? The neural correlates of everyday moral reasoning. Neuropsychologia. 2010;48(7):2018-26.

Sripada CS, Gonzalez R, Phan KL, Liberzon I. The neural correlates of intertemporal decision-making: contributions of subjective value, stimulus type, and trait impulsivity. Human brain mapping. 2011;32(10):1637-48.

van Leijenhorst L, Crone EA, Bunge SA. Neural correlates of developmental differences in risk estimation and feedback processing. Neuropsychologia. 2006;44(11):2158-70.

Wesley MJ, Hanlon CA, Porrino LJ. Poor decision-making by chronic marijuana users is associated with decreased functional responsiveness to negative consequences. Psychiatry research. 2011;191(1):51-9.

Xu L, Liang ZY, Wang K, Li S, Jiang T. Neural mechanism of intertemporal choice: from discounting future gains to future losses. Brain research. 2009;1261:65-74.

Yarkoni T, Gray JR, Chrastil ER, Barch DM, Green L, Braver TS. Sustained neural activity associated with cognitive control during temporally extended decision making. Brain research Cognitive brain research. 2005;23(1):71-84.
