# Supplementary material for: From Phineas Gage and Monsieur Leborgne to H.M.: Revisiting Disconnection Syndromes
Source: Cereb Cortex. 2015 Aug 12;25(12):4812–27. doi: 10.1093/cercor/bhv173 (PMC4635921; doi:10.1093/cercor/bhv173)
Supplement: Supplementary Data [file supp_bhv173_bhv173supp_References2.docx]

**Supplementary References 2**: List of the 215 journal articles included in the emotion meta-analysis.

Abler B, Hofer C, Walter H, Erk S, Hoffmann H, Traue HC, et al. Habitual emotion regulation strategies and depressive symptoms in healthy subjects predict fMRI brain activation patterns related to major depression. Psychiatry research. 2010;183(2):105-13.

Adams RB, Jr., Franklin RG, Jr., Rule NO, Freeman JB, Kveraga K, Hadjikhani N, et al. Culture, gaze and the neural processing of fear expressions. Social cognitive and affective neuroscience. 2010;5(2-3):340-8.

Alba-Ferrara L, Hausmann M, Mitchell RL, Weis S. The neural correlates of emotional prosody comprehension: disentangling simple from complex emotion. PloS one. 2011;6(12):e28701.

Aleman A, Swart M. Sex differences in neural activation to facial expressions denoting contempt and disgust. PloS one. 2008;3(11):e3622.

Amting JM, Miller JE, Chow M, Mitchell DG. Getting mixed messages: the impact of conflicting social signals on the brain's target emotional response. NeuroImage. 2009;47(4):1950-9.

Anderson IM, Juhasz G, Thomas E, Downey D, McKie S, Deakin JF, et al. The effect of acute citalopram on face emotion processing in remitted depression: a pharmacoMRI study. European neuropsychopharmacology : the journal of the European College of Neuropsychopharmacology. 2011;21(1):140-8.

Baeken C, De Raedt R, Ramsey N, Van Schuerbeek P, Hermes D, Bossuyt A, et al. Amygdala responses to positively and negatively valenced baby faces in healthy female volunteers: influences of individual differences in harm avoidance. Brain research. 2009;1296:94-103.

Baeken C, Van Schuerbeek P, De Raedt R, Bossuyt A, Vanderhasselt MA, De Mey J, et al. Passively viewing negatively valenced baby faces attenuates left amygdala activity in healthy females scoring high on 'Harm Avoidance'. Neuroscience letters. 2010;478(2):97-101.

Basile B, Mancini F, Macaluso E, Caltagirone C, Frackowiak RS, Bozzali M. Deontological and altruistic guilt: evidence for distinct neurobiological substrates. Human brain mapping. 2011;32(2):229-39.

Baumgartner T, Lutz K, Schmidt CF, Jancke L. The emotional power of music: how music enhances the feeling of affective pictures. Brain research. 2006;1075(1):151-64.

Berthoz S, Grezes J, Armony JL, Passingham RE, Dolan RJ. Affective response to one's own moral violations. NeuroImage. 2006;31(2):945-50.

Bertolino A, Arciero G, Rubino V, Latorre V, De Candia M, Mazzola V, et al. Variation of human amygdala response during threatening stimuli as a function of 5'HTTLPR genotype and personality style. Biological psychiatry. 2005;57(12):1517-25.

Botzung A, Rubin DC, Miles A, Cabeza R, Labar KS. Mental hoop diaries: emotional memories of a college basketball game in rival fans. The Journal of neuroscience : the official journal of the Society for Neuroscience. 2010;30(6):2130-7.

Britton JC, Phan KL, Taylor SF, Welsh RC, Berridge KC, Liberzon I. Neural correlates of social and nonsocial emotions: An fMRI study. NeuroImage. 2006;31(1):397-409.

Britton JC, Taylor SF, Sudheimer KD, Liberzon I. Facial expressions and complex IAPS pictures: common and differential networks. NeuroImage. 2006;31(2):906-19.

Brown S, Gao X, Tisdelle L, Eickhoff SB, Liotti M. Naturalizing aesthetics: brain areas for aesthetic appraisal across sensory modalities. NeuroImage. 2011;58(1):250-8.

Bruck C, Kreifelts B, Kaza E, Lotze M, Wildgruber D. Impact of personality on the cerebral processing of emotional prosody. NeuroImage. 2011;58(1):259-68.

Bruneau EG, Pluta A, Saxe R. Distinct roles of the 'shared pain' and 'theory of mind' networks in processing others' emotional suffering. Neuropsychologia. 2012;50(2):219-31.

Budell L, Jackson P, Rainville P. Brain responses to facial expressions of pain: emotional or motor mirroring? NeuroImage. 2010;53(1):355-63.

Campbell-Sills L, Simmons AN, Lovero KL, Rochlin AA, Paulus MP, Stein MB. Functioning of neural systems supporting emotion regulation in anxiety-prone individuals. NeuroImage. 2011;54(1):689-96.

Canessa N, Motterlini M, Alemanno F, Perani D, Cappa SF. Learning from other people's experience: a neuroimaging study of decisional interactive-learning. NeuroImage. 2011;55(1):353-62.

Canessa N, Motterlini M, Di Dio C, Perani D, Scifo P, Cappa SF, et al. Understanding others' regret: a FMRI study. PloS one. 2009;4(10):e7402.

Caria A, Venuti P, de Falco S. Functional and dysfunctional brain circuits underlying emotional processing of music in autism spectrum disorders. Cerebral cortex. 2011;21(12):2838-49.

Caseras X, Mataix-Cols D, An SK, Lawrence NS, Speckens A, Giampietro V, et al. Sex differences in neural responses to disgusting visual stimuli: implications for disgust-related psychiatric disorders. Biological psychiatry. 2007;62(5):464-71.

Chaminade T, Zecca M, Blakemore SJ, Takanishi A, Frith CD, Micera S, et al. Brain response to a humanoid robot in areas implicated in the perception of human emotional gestures. PloS one. 2010;5(7):e11577.

Chua HF, Gonzalez R, Taylor SF, Welsh RC, Liberzon I. Decision-related loss: regret and disappointment. NeuroImage. 2009;47(4):2031-40.

Cikara M, Farnsworth RA, Harris LT, Fiske ST. On the wrong side of the trolley track: neural correlates of relative social valuation. Social cognitive and affective neuroscience. 2010;5(4):404-13.

Curcic-Blake B, Swart M, Aleman A. Bidirectional information flow in frontoamygdalar circuits in humans: a dynamic causal modeling study of emotional associative learning. Cerebral cortex. 2012;22(2):436-45.

Dannlowski U, Ohrmann P, Bauer J, Kugel H, Arolt V, Heindel W, et al. Amygdala reactivity predicts automatic negative evaluations for facial emotions. Psychiatry research. 2007;154(1):13-20.

Danziger N, Faillenot I, Peyron R. Can we share a pain we never felt? Neural correlates of empathy in patients with congenital insensitivity to pain. Neuron. 2009;61(2):203-12.

de Greck M, Scheidt L, Bolter AF, Frommer J, Ulrich C, Stockum E, et al. Altered brain activity during emotional empathy in somatoform disorder. Human brain mapping. 2012;33(11):2666-85.

de Greck M, Wang G, Yang X, Wang X, Northoff G, Han S. Neural substrates underlying intentional empathy. Social cognitive and affective neuroscience. 2012;7(2):135-44.

Deeley Q, Daly EM, Surguladze S, Page L, Toal F, Robertson D, et al. An event related functional magnetic resonance imaging study of facial emotion processing in Asperger syndrome. Biological psychiatry. 2007;62(3):207-17.

Denkova E, Wong G, Dolcos S, Sung K, Wang L, Coupland N, et al. The impact of anxiety-inducing distraction on cognitive performance: a combined brain imaging and personality investigation. PloS one. 2010;5(11):e14150.

Dima D, Stephan KE, Roiser JP, Friston KJ, Frangou S. Effective connectivity during processing of facial affect: evidence for multiple parallel pathways. The Journal of neuroscience : the official journal of the Society for Neuroscience. 2011;31(40):14378-85.

Dolan MC, Fullam RS. Psychopathy and functional magnetic resonance imaging blood oxygenation level-dependent responses to emotional faces in violent patients with schizophrenia. Biological psychiatry. 2009;66(6):570-7.

Dolcos F, McCarthy G. Brain systems mediating cognitive interference by emotional distraction. The Journal of neuroscience : the official journal of the Society for Neuroscience. 2006;26(7):2072-9.

Domes G, Heinrichs M, Glascher J, Buchel C, Braus DF, Herpertz SC. Oxytocin attenuates amygdala responses to emotional faces regardless of valence. Biological psychiatry. 2007;62(10):1187-90.

Duan X, Dai Q, Gong Q, Chen H. Neural mechanism of unconscious perception of surprised facial expression. NeuroImage. 2010;52(1):401-7.

Dvash J, Gilam G, Ben-Ze'ev A, Hendler T, Shamay-Tsoory SG. The envious brain: the neural basis of social comparison. Human brain mapping. 2010;31(11):1741-50.

Dyck M, Loughead J, Kellermann T, Boers F, Gur RC, Mathiak K. Cognitive versus automatic mechanisms of mood induction differentially activate left and right amygdala. NeuroImage. 2011;54(3):2503-13.

Ebisch SJ, Gallese V, Willems RM, Mantini D, Groen WB, Romani GL, et al. Altered intrinsic functional connectivity of anterior and posterior insula regions in high-functioning participants with autism spectrum disorder. Human brain mapping. 2011;32(7):1013-28.

Eippert F, Veit R, Weiskopf N, Erb M, Birbaumer N, Anders S. Regulation of emotional responses elicited by threat-related stimuli. Human brain mapping. 2007;28(5):409-23.

Eldar E, Ganor O, Admon R, Bleich A, Hendler T. Feeling the real world: limbic response to music depends on related content. Cerebral cortex. 2007;17(12):2828-40.

Erk S, Mikschl A, Stier S, Ciaramidaro A, Gapp V, Weber B, et al. Acute and sustained effects of cognitive emotion regulation in major depression. The Journal of neuroscience : the official journal of the Society for Neuroscience. 2010;30(47):15726-34.

Erk S, von Kalckreuth A, Walter H. Neural long-term effects of emotion regulation on episodic memory processes. Neuropsychologia. 2010;48(4):989-96.

Ernst M, Maheu FS, Schroth E, Hardin J, Golan LG, Cameron J, et al. Amygdala function in adolescents with congenital adrenal hyperplasia: a model for the study of early steroid abnormalities. Neuropsychologia. 2007;45(9):2104-13.

Eryilmaz H, Van De Ville D, Schwartz S, Vuilleumier P. Impact of transient emotions on functional connectivity during subsequent resting state: a wavelet correlation approach. NeuroImage. 2011;54(3):2481-91.

Fecteau S, Belin P, Joanette Y, Armony JL. Amygdala responses to nonlinguistic emotional vocalizations. NeuroImage. 2007;36(2):480-7.

Finger EC, Marsh AA, Kamel N, Mitchell DG, Blair JR. Caught in the act: the impact of audience on the neural response to morally and socially inappropriate behavior. NeuroImage. 2006;33(1):414-21.

Fitzgerald DA, Angstadt M, Jelsone LM, Nathan PJ, Phan KL. Beyond threat: amygdala reactivity across multiple expressions of facial affect. NeuroImage. 2006;30(4):1441-8.

Fitzgerald DA, Posse S, Moore GJ, Tancer ME, Nathan PJ, Phan KL. Neural correlates of internally-generated disgust via autobiographical recall: a functional magnetic resonance imaging investigation. Neuroscience letters. 2004;370(2-3):91-6.

Flores-Gutierrez EO, Diaz JL, Barrios FA, Favila-Humara R, Guevara MA, del Rio-Portilla Y, et al. Metabolic and electric brain patterns during pleasant and unpleasant emotions induced by music masterpieces. International journal of psychophysiology : official journal of the International Organization of Psychophysiology. 2007;65(1):69-84.

Freed PJ, Yanagihara TK, Hirsch J, Mann JJ. Neural mechanisms of grief regulation. Biological psychiatry. 2009;66(1):33-40.

Frewen PA, Dozois DJ, Neufeld RW, Densmore M, Stevens TK, Lanius RA. Neuroimaging social emotional processing in women: fMRI study of script-driven imagery. Social cognitive and affective neuroscience. 2011;6(3):375-92.

Friederich HC, Uher R, Brooks S, Giampietro V, Brammer M, Williams SC, et al. I'm not as slim as that girl: neural bases of body shape self-comparison to media images. NeuroImage. 2007;37(2):674-81.

Fujiwara J, Tobler PN, Taira M, Iijima T, Tsutsui K. A parametric relief signal in human ventrolateral prefrontal cortex. NeuroImage. 2009;44(3):1163-70.

Geday J, Kupers R, Gjedde A. As time goes by: temporal constraints on emotional activation of inferior medial prefrontal cortex. Cerebral cortex. 2007;17(12):2753-9.

Geday J, Ostergaard K, Gjedde A. Stimulation of subthalamic nucleus inhibits emotional activation of fusiform gyrus. NeuroImage. 2006;33(2):706-14.

Gillath O, Bunge SA, Shaver PR, Wendelken C, Mikulincer M. Attachment-style differences in the ability to suppress negative thoughts: exploring the neural correlates. NeuroImage. 2005;28(4):835-47.

Goldin PR, Hutcherson CA, Ochsner KN, Glover GH, Gabrieli JD, Gross JJ. The neural bases of amusement and sadness: a comparison of block contrast and subject-specific emotion intensity regression approaches. NeuroImage. 2005;27(1):26-36.

Goodkind MS, Sollberger M, Gyurak A, Rosen HJ, Rankin KP, Miller B, et al. Tracking emotional valence: the role of the orbitofrontal cortex. Human brain mapping. 2012;33(4):753-62.

Grezes J, Pichon S, de Gelder B. Perceiving fear in dynamic body expressions. NeuroImage. 2007;35(2):959-67.

Grimm S, Ernst J, Boesiger P, Schuepbach D, Hell D, Boeker H, et al. Increased self-focus in major depressive disorder is related to neural abnormalities in subcortical-cortical midline structures. Human brain mapping. 2009;30(8):2617-27.

Grimm S, Schmidt CF, Bermpohl F, Heinzel A, Dahlem Y, Wyss M, et al. Segregated neural representation of distinct emotion dimensions in the prefrontal cortex-an fMRI study. NeuroImage. 2006;30(1):325-40.

Habel U, Chechko N, Pauly K, Koch K, Backes V, Seiferth N, et al. Neural correlates of emotion recognition in schizophrenia. Schizophrenia research. 2010;122(1-3):113-23.

Habel U, Klein M, Kellermann T, Shah NJ, Schneider F. Same or different? Neural correlates of happy and sad mood in healthy males. NeuroImage. 2005;26(1):206-14.

Habel U, Windischberger C, Derntl B, Robinson S, Kryspin-Exner I, Gur RC, et al. Amygdala activation and facial expressions: explicit emotion discrimination versus implicit emotion processing. Neuropsychologia. 2007;45(10):2369-77.

Hadjikhani N, Joseph RM, Manoach DS, Naik P, Snyder J, Dominick K, et al. Body expressions of emotion do not trigger fear contagion in autism spectrum disorder. Social cognitive and affective neuroscience. 2009;4(1):70-8.

Hall J, Whalley HC, McKirdy JW, Romaniuk L, McGonigle D, McIntosh AM, et al. Overactivation of fear systems to neutral faces in schizophrenia. Biological psychiatry. 2008;64(1):70-3.

Han S, Qin J, Ma Y. Neurocognitive processes of linguistic cues related to death. Neuropsychologia. 2010;48(12):3436-42.

Harenski CL, Hamann S. Neural correlates of regulating negative emotions related to moral violations. NeuroImage. 2006;30(1):313-24.

Harris LT, Fiske ST. Social groups that elicit disgust are differentially processed in mPFC. Social cognitive and affective neuroscience. 2007;2(1):45-51.

Herbert C, Herbert BM, Pauli P. Emotional self-reference: brain structures involved in the processing of words describing one's own emotions. Neuropsychologia. 2011;49(10):2947-56.

Hermann A, Schafer A, Walter B, Stark R, Vaitl D, Schienle A. Emotion regulation in spider phobia: role of the medial prefrontal cortex. Social cognitive and affective neuroscience. 2009;4(3):257-67.

Herwig U, Kaffenberger T, Jancke L, Bruhl AB. Self-related awareness and emotion regulation. NeuroImage. 2010;50(2):734-41.

Hofer A, Siedentopf CM, Ischebeck A, Rettenbacher MA, Verius M, Felber S, et al. Gender differences in regional cerebral activity during the perception of emotion: a functional MRI study. NeuroImage. 2006;32(2):854-62.

Hooker CI, Verosky SC, Germine LT, Knight RT, D'Esposito M. Mentalizing about emotion and its relationship to empathy. Social cognitive and affective neuroscience. 2008;3(3):204-17.

Hutcherson CA, Goldin PR, Ramel W, McRae K, Gross JJ. Attention and emotion influence the relationship between extraversion and neural response. Social cognitive and affective neuroscience. 2008;3(1):71-9.

Hutcherson CA, Gross JJ. The moral emotions: a social-functionalist account of anger, disgust, and contempt. Journal of personality and social psychology. 2011;100(4):719-37.

Iaria G, Committeri G, Pastorelli C, Pizzamiglio L, Watkins KE, Carota A. Neural activity of the anterior insula in emotional processing depends on the individuals' emotional susceptibility. Human brain mapping. 2008;29(3):363-73.

Ibarretxe-Bilbao N, Junque C, Tolosa E, Marti MJ, Valldeoriola F, Bargallo N, et al. Neuroanatomical correlates of impaired decision-making and facial emotion recognition in early Parkinson's disease. The European journal of neuroscience. 2009;30(6):1162-71.

Immordino-Yang MH, Singh V. Hippocampal contributions to the processing of social emotions. Human brain mapping. 2013;34(4):945-55.

Ioannides AA, Liu LC, Kwapien J, Drozdz S, Streit M. Coupling of regional activations in a human brain during an object and face affect recognition task. Human brain mapping. 2000;11(2):77-92.

Jabbi M, Bastiaansen J, Keysers C. A common anterior insula representation of disgust observation, experience and imagination shows divergent functional connectivity pathways. PloS one. 2008;3(8):e2939.

Jehna M, Neuper C, Ischebeck A, Loitfelder M, Ropele S, Langkammer C, et al. The functional correlates of face perception and recognition of emotional facial expressions as evidenced by fMRI. Brain research. 2011;1393:73-83.

Jimura K, Konishi S, Miyashita Y. Temporal pole activity during perception of sad faces, but not happy faces, correlates with neuroticism trait. Neuroscience letters. 2009;453(1):45-8.

Johnston PJ, Stojanov W, Devir H, Schall U. Functional MRI of facial emotion recognition deficits in schizophrenia and their electrophysiological correlates. The European journal of neuroscience. 2005;22(5):1221-32.

Johnstone T, van Reekum CM, Oakes TR, Davidson RJ. The voice of emotion: an FMRI study of neural responses to angry and happy vocal expressions. Social cognitive and affective neuroscience. 2006;1(3):242-9.

Jung HT, Kim DW, Kim S, Im CH, Lee SH. Reduced source activity of event-related potentials for affective facial pictures in schizophrenia patients. Schizophrenia research. 2012;136(1-3):150-9.

Kana RK, Travers BG. Neural substrates of interpreting actions and emotions from body postures. Social cognitive and affective neuroscience. 2012;7(4):446-56.

Keightley ML, Chiew KS, Winocur G, Grady CL. Age-related differences in brain activity underlying identification of emotional expressions in faces. Social cognitive and affective neuroscience. 2007;2(4):292-302.

Killgore WD, Yurgelun-Todd DA. The right-hemisphere and valence hypotheses: could they both be right (and sometimes left)? Social cognitive and affective neuroscience. 2007;2(3):240-50.

Kim JW, Choi EA, Kim JJ, Jeong BS, Kim SE, Ki SW. The role of amygdala during auditory verbal imagery of derogatory appraisals by others. Neuroscience letters. 2008;446(1):1-6.

Kim JW, Kim SE, Kim JJ, Jeong B, Park CH, Son AR, et al. Compassionate attitude towards others' suffering activates the mesolimbic neural system. Neuropsychologia. 2009;47(10):2073-81.

Kim SE, Kim JW, Kim JJ, Jeong BS, Choi EA, Jeong YG, et al. The neural mechanism of imagining facial affective expression. Brain research. 2007;1145:128-37.

Klasen M, Kenworthy CA, Mathiak KA, Kircher TT, Mathiak K. Supramodal representation of emotions. The Journal of neuroscience : the official journal of the Society for Neuroscience. 2011;31(38):13635-43.

Koelsch S, Fritz T, DY VC, Muller K, Friederici AD. Investigating emotion with music: an fMRI study. Human brain mapping. 2006;27(3):239-50.

Kohn N, Kellermann T, Gur RC, Schneider F, Habel U. Gender differences in the neural correlates of humor processing: implications for different processing modes. Neuropsychologia. 2011;49(5):888-97.

Koven NS, Roth RM, Garlinghouse MA, Flashman LA, Saykin AJ. Regional gray matter correlates of perceived emotional intelligence. Social cognitive and affective neuroscience. 2011;6(5):582-90.

Krach S, Cohrs JC, de Echeverria Loebell NC, Kircher T, Sommer J, Jansen A, et al. Your flaws are my pain: linking empathy to vicarious embarrassment. PloS one. 2011;6(4):e18675.

Kramer UM, Mohammadi B, Donamayor N, Samii A, Munte TF. Emotional and cognitive aspects of empathy and their relation to social cognition--an fMRI-study. Brain research. 2010;1311:110-20.

Krendl AC, Kensinger EA, Ambady N. How does the brain regulate negative bias to stigma? Social cognitive and affective neuroscience. 2012;7(6):715-26.

Kret ME, Denollet J, Grezes J, de Gelder B. The role of negative affectivity and social inhibition in perceiving social threat: an fMRI study. Neuropsychologia. 2011;49(5):1187-93.

Lane RD, McRae K, Reiman EM, Chen K, Ahern GL, Thayer JF. Neural correlates of heart rate variability during emotion. NeuroImage. 2009;44(1):213-22.

Lang S, Kotchoubey B, Frick C, Spitzer C, Grabe HJ, Barnow S. Cognitive reappraisal in trauma-exposed women with borderline personality disorder. NeuroImage. 2012;59(2):1727-34.

Lanius RA, Williamson PC, Bluhm RL, Densmore M, Boksman K, Neufeld RW, et al. Functional connectivity of dissociative responses in posttraumatic stress disorder: a functional magnetic resonance imaging investigation. Biological psychiatry. 2005;57(8):873-84.

Lau JY, Goldman D, Buzas B, Fromm SJ, Guyer AE, Hodgkinson C, et al. Amygdala function and 5-HTT gene variants in adolescent anxiety and major depressive disorder. Biological psychiatry. 2009;65(4):349-55.

Lee BT, Lee HY, Han C, Pae CU, Tae WS, Lee MS, et al. DRD2/ANKK1 TaqI A polymorphism affects corticostriatal activity in response to negative affective facial stimuli. Behavioural brain research. 2011;223(1):36-41.

Lee KH, Siegle GJ. Common and distinct brain networks underlying explicit emotional evaluation: a meta-analytic study. Social cognitive and affective neuroscience. 2012;7(5):521-34.

Lee TM, Lee TM, Raine A, Chan CC. Lying about the valence of affective pictures: an fMRI study. PloS one. 2010;5(8):e12291.

Lee TW, Dolan RJ, Critchley HD. Controlling emotional expression: behavioral and neural correlates of nonimitative emotional responses. Cerebral cortex. 2008;18(1):104-13.

Lenzi D, Trentini C, Pantano P, Macaluso E, Iacoboni M, Lenzi GL, et al. Neural basis of maternal communication and emotional expression processing during infant preverbal stage. Cerebral cortex. 2009;19(5):1124-33.

Lerner A, Bagic A, Hanakawa T, Boudreau EA, Pagan F, Mari Z, et al. Involvement of insula and cingulate cortices in control and suppression of natural urges. Cerebral cortex. 2009;19(1):218-23.

Levesque J, Joanette Y, Mensour B, Beaudoin G, Leroux JM, Bourgouin P, et al. Neural basis of emotional self-regulation in childhood. Neuroscience. 2004;129(2):361-9.

Li Q, Qin S, Rao LL, Zhang W, Ying X, Guo X, et al. Can Sophie's choice be adequately captured by cold computation of minimizing losses? An fMRI study of vital loss decisions. PloS one. 2011;6(3):e17544.

Liu L, Ioannides AA. Emotion separation is completed early and it depends on visual field presentation. PloS one. 2010;5(3):e9790.

Longe O, Maratos FA, Gilbert P, Evans G, Volker F, Rockliff H, et al. Having a word with yourself: neural correlates of self-criticism and self-reassurance. NeuroImage. 2010;49(2):1849-56.

Lotze M, Veit R, Anders S, Birbaumer N. Evidence for a different role of the ventral and dorsal medial prefrontal cortex for social reactive aggression: An interactive fMRI study. NeuroImage. 2007;34(1):470-8.

Loughead J, Gur RC, Elliott M, Gur RE. Neural circuitry for accurate identification of facial emotions. Brain research. 2008;1194:37-44.

Mak AK, Hu ZG, Zhang JX, Xiao ZW, Lee TM. Neural correlates of regulation of positive and negative emotions: an fmri study. Neuroscience letters. 2009;457(2):101-6.

Mantani T, Okamoto Y, Shirao N, Okada G, Yamawaki S. Reduced activation of posterior cingulate cortex during imagery in subjects with high degrees of alexithymia: a functional magnetic resonance imaging study. Biological psychiatry. 2005;57(9):982-90.

Matsunaga M, Isowa T, Kimura K, Miyakoshi M, Kanayama N, Murakami H, et al. Associations among positive mood, brain, and cardiovascular activities in an affectively positive situation. Brain research. 2009;1263:93-103.

Mazzola V, Latorre V, Petito A, Gentili N, Fazio L, Popolizio T, et al. Affective response to a loved one's pain: insula activity as a function of individual differences. PloS one. 2010;5(12):e15268.

Mendrek A, Jimenez JA, Mancini-Marie A, Fahim C, Stip E. Correlations between sadness-induced cerebral activations and schizophrenia symptoms: an fMRI study of sex differences. European psychiatry : the journal of the Association of European Psychiatrists. 2011;26(5):320-6.

Mercadillo RE, Diaz JL, Pasaye EH, Barrios FA. Perception of suffering and compassion experience: brain gender disparities. Brain and cognition. 2011;76(1):5-14.

Meriau K, Wartenburger I, Kazzer P, Prehn K, Lammers CH, van der Meer E, et al. A neural network reflecting individual differences in cognitive processing of emotions during perceptual decision making. NeuroImage. 2006;33(3):1016-27.

Meriau K, Wartenburger I, Kazzer P, Prehn K, Villringer A, van der Meer E, et al. Insular activity during passive viewing of aversive stimuli reflects individual differences in state negative affect. Brain and cognition. 2009;69(1):73-80.

Mitchell RL, Ross ED. fMRI evidence for the effect of verbal complexity on lateralisation of the neural response associated with decoding prosodic emotion. Neuropsychologia. 2008;46(12):2880-7.

Miyahara M, Harada T, Ruffman T, Sadato N, Iidaka T. Functional connectivity between amygdala and facial regions involved in recognition of facial threat. Social cognitive and affective neuroscience. 2013;8(2):181-9.

Mocaiber I, Sanchez TA, Pereira MG, Erthal FS, Joffily M, Araujo DB, et al. Antecedent descriptions change brain reactivity to emotional stimuli: a functional magnetic resonance imaging study of an extrinsic and incidental reappraisal strategy. Neuroscience. 2011;193:241-8.

Modinos G, Ormel J, Aleman A. Individual differences in dispositional mindfulness and brain activity involved in reappraisal of emotion. Social cognitive and affective neuroscience. 2010;5(4):369-77.

Montag C, Reuter M, Axmacher N. How one's favorite song activates the reward circuitry of the brain: personality matters! Behavioural brain research. 2011;225(2):511-4.

Moriguchi Y, Ohnishi T, Lane RD, Maeda M, Mori T, Nemoto K, et al. Impaired self-awareness and theory of mind: an fMRI study of mentalizing in alexithymia. NeuroImage. 2006;32(3):1472-82.

Morris JD, Klahr NJ, Shen F, Villegas J, Wright P, He G, et al. Mapping a multidimensional emotion in response to television commercials. Human brain mapping. 2009;30(3):789-96.

Moseley R, Carota F, Hauk O, Mohr B, Pulvermuller F. A role for the motor system in binding abstract emotional meaning. Cerebral cortex. 2012;22(7):1634-47.

Mowrer SM, Jahn AA, Abduljalil A, Cunningham WA. The value of success: acquiring gains, avoiding losses, and simply being successful. PloS one. 2011;6(9):e25307.

Mukherjee P, Whalley HC, McKirdy JW, McIntosh AM, Johnstone EC, Lawrie SM, et al. Effects of the BDNF Val66Met polymorphism on neural responses to facial emotion. Psychiatry research. 2011;191(3):182-8.

Noriuchi M, Kikuchi Y, Senoo A. The functional neuroanatomy of maternal love: mother's response to infant's attachment behaviors. Biological psychiatry. 2008;63(4):415-23.

Northoff G, Richter A, Gessner M, Schlagenhauf F, Fell J, Baumgart F, et al. Functional dissociation between medial and lateral prefrontal cortical spatiotemporal activation in negative and positive emotions: a combined fMRI/MEG study. Cerebral cortex. 2000;10(1):93-107.

Nummenmaa L, Hirvonen J, Parkkola R, Hietanen JK. Is emotional contagion special? An fMRI study on neural systems for affective and cognitive empathy. NeuroImage. 2008;43(3):571-80.

Ogino Y, Nemoto H, Inui K, Saito S, Kakigi R, Goto F. Inner experience of pain: imagination of pain while viewing images showing painful events forms subjective pain representation in human brain. Cerebral cortex. 2007;17(5):1139-46.

Ohira H, Nomura M, Ichikawa N, Isowa T, Iidaka T, Sato A, et al. Association of neural and physiological responses during voluntary emotion suppression. NeuroImage. 2006;29(3):721-33.

Omar R, Henley SM, Bartlett JW, Hailstone JC, Gordon E, Sauter DA, et al. The structural neuroanatomy of music emotion recognition: evidence from frontotemporal lobar degeneration. NeuroImage. 2011;56(3):1814-21.

Park IH, Park HJ, Chun JW, Kim EY, Kim JJ. Dysfunctional modulation of emotional interference in the medial prefrontal cortex in patients with schizophrenia. Neuroscience letters. 2008;440(2):119-24.

Park JY, Gu BM, Kang DH, Shin YW, Choi CH, Lee JM, et al. Integration of cross-modal emotional information in the human brain: an fMRI study. Cortex; a journal devoted to the study of the nervous system and behavior. 2010;46(2):161-9.

Passarotti AM, Sweeney JA, Pavuluri MN. Neural correlates of incidental and directed facial emotion processing in adolescents and adults. Social cognitive and affective neuroscience. 2009;4(4):387-98.

Pavuluri MN, Passarotti AM, Lu LH, Carbray JA, Sweeney JA. Double-blind randomized trial of risperidone versus divalproex in pediatric bipolar disorder: fMRI outcomes. Psychiatry research. 2011;193(1):28-37.

Peelen MV, Atkinson AP, Andersson F, Vuilleumier P. Emotional modulation of body-selective visual areas. Social cognitive and affective neuroscience. 2007;2(4):274-83.

Peelen MV, Atkinson AP, Vuilleumier P. Supramodal representations of perceived emotions in the human brain. The Journal of neuroscience : the official journal of the Society for Neuroscience. 2010;30(30):10127-34.

Pelphrey KA, Morris JP, McCarthy G, Labar KS. Perception of dynamic changes in facial affect and identity in autism. Social cognitive and affective neuroscience. 2007;2(2):140-9.

Pereira CS, Teixeira J, Figueiredo P, Xavier J, Castro SL, Brattico E. Music and emotions in the brain: familiarity matters. PloS one. 2011;6(11):e27241.

Petrini K, Crabbe F, Sheridan C, Pollick FE. The music of your emotions: neural substrates involved in detection of emotional correspondence between auditory and visual music actions. PloS one. 2011;6(4):e19165.

Pfeifer JH, Iacoboni M, Mazziotta JC, Dapretto M. Mirroring others' emotions relates to empathy and interpersonal competence in children. NeuroImage. 2008;39(4):2076-85.

Pfeifer JH, Masten CL, Moore WE, 3rd, Oswald TM, Mazziotta JC, Iacoboni M, et al. Entering adolescence: resistance to peer influence, risky behavior, and neural changes in emotion reactivity. Neuron. 2011;69(5):1029-36.

Phan KL, Fitzgerald DA, Nathan PJ, Moore GJ, Uhde TW, Tancer ME. Neural substrates for voluntary suppression of negative affect: a functional magnetic resonance imaging study. Biological psychiatry. 2005;57(3):210-9.

Pollatos O, Schandry R, Auer DP, Kaufmann C. Brain structures mediating cardiovascular arousal and interoceptive awareness. Brain research. 2007;1141:178-87.

Posner J, Russell JA, Gerber A, Gorman D, Colibazzi T, Yu S, et al. The neurophysiological bases of emotion: An fMRI study of the affective circumplex using emotion-denoting words. Human brain mapping. 2009;30(3):883-95.

Pouga L, Berthoz S, de Gelder B, Grezes J. Individual differences in socioaffective skills influence the neural bases of fear processing: the case of alexithymia. Human brain mapping. 2010;31(10):1469-81.

Prehn-Kristensen A, Wiesner C, Bergmann TO, Wolff S, Jansen O, Mehdorn HM, et al. Induction of empathy by the smell of anxiety. PloS one. 2009;4(6):e5987.

Rauch AV, Ohrmann P, Bauer J, Kugel H, Engelien A, Arolt V, et al. Cognitive coping style modulates neural responses to emotional faces in healthy humans: a 3-T FMRI study. Cerebral cortex. 2007;17(11):2526-35.

Reker M, Ohrmann P, Rauch AV, Kugel H, Bauer J, Dannlowski U, et al. Individual differences in alexithymia and brain response to masked emotion faces. Cortex; a journal devoted to the study of the nervous system and behavior. 2010;46(5):658-67.

Rilling JK, Glenn AL, Jairam MR, Pagnoni G, Goldsmith DR, Elfenbein HA, et al. Neural correlates of social cooperation and non-cooperation as a function of psychopathy. Biological psychiatry. 2007;61(11):1260-71.

Robins DL, Hunyadi E, Schultz RT. Superior temporal activation in response to dynamic audio-visual emotional cues. Brain and cognition. 2009;69(2):269-78.

Roder CH, Mohr H, Linden DE. Retention of identity versus expression of emotional faces differs in the recruitment of limbic areas. Neuropsychologia. 2011;49(3):444-53.

Rohrer JD, Sauter D, Scott S, Rossor MN, Warren JD. Receptive prosody in nonfluent primary progressive aphasias. Cortex; a journal devoted to the study of the nervous system and behavior. 2012;48(3):308-16.

Rosen HJ, Wilson MR, Schauer GF, Allison S, Gorno-Tempini ML, Pace-Savitsky C, et al. Neuroanatomical correlates of impaired recognition of emotion in dementia. Neuropsychologia. 2006;44(3):365-73.

Ruz M, Tudela P. Emotional conflict in interpersonal interactions. NeuroImage. 2011;54(2):1685-91.

Sambataro F, Dimalta S, Di Giorgio A, Taurisano P, Blasi G, Scarabino T, et al. Preferential responses in amygdala and insula during presentation of facial contempt and disgust. The European journal of neuroscience. 2006;24(8):2355-62.

Sass K, Habel U, Sachs O, Huber W, Gauggel S, Kircher T. The influence of emotional associations on the neural correlates of semantic priming. Human brain mapping. 2012;33(3):676-94.

Sato W, Kochiyama T, Uono S, Yoshikawa S. Amygdala integrates emotional expression and gaze direction in response to dynamic facial expressions. NeuroImage. 2010;50(4):1658-65.

Schafer A, Schienle A, Vaitl D. Stimulus type and design influence hemodynamic responses towards visual disgust and fear elicitors. International journal of psychophysiology : official journal of the International Organization of Psychophysiology. 2005;57(1):53-9.

Schardt DM, Erk S, Nusser C, Nothen MM, Cichon S, Rietschel M, et al. Volition diminishes genetically mediated amygdala hyperreactivity. NeuroImage. 2010;53(3):943-51.

Schlund MW, Cataldo MF. Amygdala involvement in human avoidance, escape and approach behavior. NeuroImage. 2010;53(2):769-76.

Schroeder U, Hennenlotter A, Erhard P, Haslinger B, Stahl R, Lange KW, et al. Functional neuroanatomy of perceiving surprised faces. Human brain mapping. 2004;23(4):181-7.

Schulte-Ruther M, Markowitsch HJ, Shah NJ, Fink GR, Piefke M. Gender differences in brain networks supporting empathy. NeuroImage. 2008;42(1):393-403.

Schulze L, Domes G, Kruger A, Berger C, Fleischer M, Prehn K, et al. Neuronal correlates of cognitive reappraisal in borderline patients with affective instability. Biological psychiatry. 2011;69(6):564-73.

Seiferth NY, Pauly K, Habel U, Kellermann T, Shah NJ, Ruhrmann S, et al. Increased neural response related to neutral faces in individuals at risk for psychosis. NeuroImage. 2008;40(1):289-97.

Seitz RJ, Schafer R, Scherfeld D, Friederichs S, Popp K, Wittsack HJ, et al. Valuating other people's emotional face expression: a combined functional magnetic resonance imaging and electroencephalography study. Neuroscience. 2008;152(3):713-22.

Simon D, Craig KD, Miltner WH, Rainville P. Brain responses to dynamic facial expressions of pain. Pain. 2006;126(1-3):309-18.

Simon-Thomas ER, Godzik J, Castle E, Antonenko O, Ponz A, Kogan A, et al. An fMRI study of caring vs self-focus during induced compassion and pride. Social cognitive and affective neuroscience. 2012;7(6):635-48.

Sommer M, Dohnel K, Meinhardt J, Hajak G. Decoding of affective facial expressions in the context of emotional situations. Neuropsychologia. 2008;46(11):2615-21.

Sommer M, Rothmayr C, Dohnel K, Meinhardt J, Schwerdtner J, Sodian B, et al. How should I decide? The neural correlates of everyday moral reasoning. Neuropsychologia. 2010;48(7):2018-26.

Sreenivas S, Boehm SG, Linden DE. Emotional faces and the default mode network. Neuroscience letters. 2012;506(2):229-34.

St Jacques P, Dolcos F, Cabeza R. Effects of aging on functional connectivity of the amygdala during negative evaluation: a network analysis of fMRI data. Neurobiology of aging. 2010;31(2):315-27.

Stark R, Schienle A, Sarlo M, Palomba D, Walter B, Vaitl D. Influences of disgust sensitivity on hemodynamic responses towards a disgust-inducing film clip. International journal of psychophysiology : official journal of the International Organization of Psychophysiology. 2005;57(1):61-7.

Steele JD, Meyer M, Ebmeier KP. Neural predictive error signal correlates with depressive illness severity in a game paradigm. NeuroImage. 2004;23(1):269-80.

Suslow T, Kugel H, Rauch AV, Dannlowski U, Bauer J, Konrad C, et al. Attachment avoidance modulates neural response to masked facial emotion. Human brain mapping. 2009;30(11):3553-62.

Suslow T, Kugel H, Reber H, Bauer J, Dannlowski U, Kersting A, et al. Automatic brain response to facial emotion as a function of implicitly and explicitly measured extraversion. Neuroscience. 2010;167(1):111-23.

Takahashi H, Kato M, Matsuura M, Koeda M, Yahata N, Suhara T, et al. Neural correlates of human virtue judgment. Cerebral cortex. 2008;18(8):1886-91.

Takahashi H, Matsuura M, Koeda M, Yahata N, Suhara T, Kato M, et al. Brain activations during judgments of positive self-conscious emotion and positive basic emotion: pride and joy. Cerebral cortex. 2008;18(4):898-903.

Takahashi H, Matsuura M, Yahata N, Koeda M, Suhara T, Okubo Y. Men and women show distinct brain activations during imagery of sexual and emotional infidelity. NeuroImage. 2006;32(3):1299-307.

Takahashi H, Yahata N, Koeda M, Matsuda T, Asai K, Okubo Y. Brain activation associated with evaluative processes of guilt and embarrassment: an fMRI study. NeuroImage. 2004;23(3):967-74.

Thornton-Wells TA, Avery SN, Blackford JU. Using novel control groups to dissect the amygdala's role in Williams syndrome. Developmental cognitive neuroscience. 2011;1(3):295-304.

Trost W, Ethofer T, Zentner M, Vuilleumier P. Mapping aesthetic musical emotions in the brain. Cerebral cortex. 2012;22(12):2769-83.

Ushida T, Ikemoto T, Tanaka S, Shinozaki J, Taniguchi S, Murata Y, et al. Virtual needle pain stimuli activates cortical representation of emotions in normal volunteers. Neuroscience letters. 2008;439(1):7-12.

Viinikainen M, Jaaskelainen IP, Alexandrov Y, Balk MH, Autti T, Sams M. Nonlinear relationship between emotional valence and brain activity: evidence of separate negative and positive valence dimensions. Human brain mapping. 2010;31(7):1030-40.

Vilhauer RP. 'Them' and 'us': the experiences of women with metastatic disease in mixed-stage versus stage-specific breast cancer support groups. Psychology & health. 2011;26(6):781-97.

Vocks S, Busch M, Schulte D, Gronermeyer D, Herpertz S, Suchan B. Effects of body image therapy on the activation of the extrastriate body area in anorexia nervosa: an fMRI study. Psychiatry research. 2010;183(2):114-8.

Vollm BA, Taylor AN, Richardson P, Corcoran R, Stirling J, McKie S, et al. Neuronal correlates of theory of mind and empathy: a functional magnetic resonance imaging study in a nonverbal task. NeuroImage. 2006;29(1):90-8.

von dem Hagen EA, Beaver JD, Ewbank MP, Keane J, Passamonti L, Lawrence AD, et al. Leaving a bad taste in your mouth but not in my insula. Social cognitive and affective neuroscience. 2009;4(4):379-86.

Vrticka P, Sander D, Vuilleumier P. Effects of emotion regulation strategy on brain responses to the valence and social content of visual scenes. Neuropsychologia. 2011;49(5):1067-82.

Wagner U, N'Diaye K, Ethofer T, Vuilleumier P. Guilt-specific processing in the prefrontal cortex. Cerebral cortex. 2011;21(11):2461-70.

Wallentin M, Nielsen AH, Vuust P, Dohn A, Roepstorff A, Lund TE. Amygdala and heart rate variability responses from listening to emotionally intense parts of a story. NeuroImage. 2011;58(3):963-73.

Wang DJ, Rao H, Korczykowski M, Wintering N, Pluta J, Khalsa DS, et al. Cerebral blood flow changes associated with different meditation practices and perceived depth of meditation. Psychiatry research. 2011;191(1):60-7.

Waugh CE, Hamilton JP, Gotlib IH. The neural temporal dynamics of the intensity of emotional experience. NeuroImage. 2010;49(2):1699-707.

Waugh CE, Wager TD, Fredrickson BL, Noll DC, Taylor SF. The neural correlates of trait resilience when anticipating and recovering from threat. Social cognitive and affective neuroscience. 2008;3(4):322-32.

Wei L, Duan X, Yang Y, Liao W, Gao Q, Ding JR, et al. The synchronization of spontaneous BOLD activity predicts extraversion and neuroticism. Brain research. 2011;1419:68-75.

Welborn BL, Papademetris X, Reis DL, Rajeevan N, Bloise SM, Gray JR. Variation in orbitofrontal cortex volume: relation to sex, emotion regulation and affect. Social cognitive and affective neuroscience. 2009;4(4):328-39.

Weniger G, Irle E. Impaired facial affect recognition and emotional changes in subjects with transmodal cortical lesions. Cerebral cortex. 2002;12(3):258-68.

Wildgruber D, Riecker A, Hertrich I, Erb M, Grodd W, Ethofer T, et al. Identification of emotional intonation evaluated by fMRI. NeuroImage. 2005;24(4):1233-41.

Wilson-Mendenhall CD, Barrett LF, Simmons WK, Barsalou LW. Grounding emotion in situated conceptualization. Neuropsychologia. 2011;49(5):1105-27.

Winecoff A, Labar KS, Madden DJ, Cabeza R, Huettel SA. Cognitive and neural contributors to emotion regulation in aging. Social cognitive and affective neuroscience. 2011;6(2):165-76.

Xu L, Liang ZY, Wang K, Li S, Jiang T. Neural mechanism of intertemporal choice: from discounting future gains to future losses. Brain research. 2009;1261:65-74.

Yamada M, Hirao K, Namiki C, Hanakawa T, Fukuyama H, Hayashi T, et al. Social cognition and frontal lobe pathology in schizophrenia: a voxel-based morphometric study. NeuroImage. 2007;35(1):292-8.
