# Supplementary material for: From Phineas Gage and Monsieur Leborgne to H.M.: Revisiting Disconnection Syndromes
Source: Cereb Cortex. 2015 Aug 12;25(12):4812–27. doi: 10.1093/cercor/bhv173 (PMC4635921; doi:10.1093/cercor/bhv173)
Supplement: Supplementary Data [file supp_bhv173_bhv173supp_References3.docx]

**Supplementary References 3**: List of the 75 journal articles included in the fluency meta-analysis.

Abrahams S, Goldstein LH, Simmons A, Brammer MJ, Williams SC, Giampietro VP, et al. Functional magnetic resonance imaging of verbal fluency and confrontation naming using compressed image acquisition to permit overt responses. Human brain mapping. 2003;20(1):29-40.

Allendorfer JB, Lindsell CJ, Siegel M, Banks CL, Vannest J, Holland SK, et al. Females and males are highly similar in language performance and cortical activation patterns during verb generation. Cortex; a journal devoted to the study of the nervous system and behavior. 2012;48(9):1218-33.

Ash S, McMillan C, Gross RG, Cook P, Gunawardena D, Morgan B, et al. Impairments of speech fluency in Lewy body spectrum disorder. Brain and language. 2012;120(3):290-302.

Ash S, McMillan C, Gross RG, Cook P, Morgan B, Boller A, et al. The organization of narrative discourse in Lewy body spectrum disorder. Brain and language. 2011;119(1):30-41.

Balsamo LM, Xu B, Gaillard WD. Language lateralization and the role of the fusiform gyrus in semantic processing in young children. NeuroImage. 2006;31(3):1306-14.

Basho S, Palmer ED, Rubio MA, Wulfeck B, Muller RA. Effects of generation mode in fMRI adaptations of semantic fluency: paced production and overt speech. Neuropsychologia. 2007;45(8):1697-706.

Bava S, Thayer R, Jacobus J, Ward M, Jernigan TL, Tapert SF. Longitudinal characterization of white matter maturation during adolescence. Brain research. 2010;1327:38-46.

Benjamin CF, Gaab N. What's the story? The tale of reading fluency told at speed. Human brain mapping. 2012;33(11):2572-85.

Boksman K, Theberge J, Williamson P, Drost DJ, Malla A, Densmore M, et al. A 4.0-T fMRI study of brain connectivity during word fluency in first-episode schizophrenia. Schizophrenia research. 2005;75(2-3):247-63.

Bonelli SB, Powell R, Thompson PJ, Yogarajah M, Focke NK, Stretton J, et al. Hippocampal activation correlates with visual confrontation naming: fMRI findings in controls and patients with temporal lobe epilepsy. Epilepsy research. 2011;95(3):246-54.

Borovsky A, Saygin AP, Bates E, Dronkers N. Lesion correlates of conversational speech production deficits. Neuropsychologia. 2007;45(11):2525-33.

Chavez-Eakle RA, Graff-Guerrero A, Garcia-Reyna JC, Vaugier V, Cruz-Fuentes C. Cerebral blood flow associated with creative performance: a comparative study. NeuroImage. 2007;38(3):519-28.

Chow ML, Brambati SM, Gorno-Tempini ML, Miller BL, Johnson JK. Sound naming in neurodegenerative disease. Brain and cognition. 2010;72(3):423-9.

Costafreda SG, Fu CH, Lee L, Everitt B, Brammer MJ, David AS. A systematic review and quantitative appraisal of fMRI studies of verbal fluency: role of the left inferior frontal gyrus. Human brain mapping. 2006;27(10):799-810.

Costafreda SG, Fu CH, Picchioni M, Kane F, McDonald C, Prata DP, et al. Increased inferior frontal activation during word generation: a marker of genetic risk for schizophrenia but not bipolar disorder? Human brain mapping. 2009;30(10):3287-98.

Crinion JT, Green DW, Chung R, Ali N, Grogan A, Price GR, et al. Neuroanatomical markers of speaking Chinese. Human brain mapping. 2009;30(12):4108-15.

De Smedt B, Holloway ID, Ansari D. Effects of problem size and arithmetic operation on brain activation during calculation in children with varying levels of arithmetical fluency. NeuroImage. 2011;57(3):771-81.

de Souza LC, Volle E, Bertoux M, Czernecki V, Funkiewiez A, Allali G, et al. Poor creativity in frontotemporal dementia: a window into the neural bases of the creative mind. Neuropsychologia. 2010;48(13):3733-42.

de Zubicaray GI, Rose SE, McMahon KL. The structure and connectivity of semantic memory in the healthy older adult brain. NeuroImage. 2011;54(2):1488-94.

Dickey CC, Morocz IA, Minney D, Niznikiewicz MA, Voglmaier MM, Panych LP, et al. Factors in sensory processing of prosody in schizotypal personality disorder: an fMRI experiment. Schizophrenia research. 2010;121(1-3):75-89.

Donnelly KM, Allendorfer JB, Szaflarski JP. Right hemispheric participation in semantic decision improves performance. Brain research. 2011;1419:105-16.

Fox PT, Ingham RJ, Ingham JC, Zamarripa F, Xiong JH, Lancaster JL. Brain correlates of stuttering and syllable production. A PET performance-correlation analysis. Brain : a journal of neurology. 2000;123 ( Pt 10):1985-2004.

Fusar-Poli P, Broome MR, Matthiasson P, Williams SC, Brammer M, McGuire PK. Effects of acute antipsychotic treatment on brain activation in first episode psychosis: an fMRI study. European neuropsychopharmacology : the journal of the European College of Neuropsychopharmacology. 2007;17(6-7):492-500.

Gaillard WD, Sachs BC, Whitnah JR, Ahmad Z, Balsamo LM, Petrella JR, et al. Developmental aspects of language processing: fMRI of verbal fluency in children and adults. Human brain mapping. 2003;18(3):176-85.

Gauthier CT, Duyme M, Zanca M, Capron C. Sex and performance level effects on brain activation during a verbal fluency task: a functional magnetic resonance imaging study. Cortex; a journal devoted to the study of the nervous system and behavior. 2009;45(2):164-76.

Goldstein RZ, Tomasi D, Alia-Klein N, Honorio Carrillo J, Maloney T, Woicik PA, et al. Dopaminergic response to drug words in cocaine addiction. The Journal of neuroscience : the official journal of the Society for Neuroscience. 2009;29(18):6001-6.

Grogan A, Green DW, Ali N, Crinion JT, Price CJ. Structural correlates of semantic and phonemic fluency ability in first and second languages. Cerebral cortex. 2009;19(11):2690-8.

Grol MJ, de Lange FP, Verstraten FA, Passingham RE, Toni I. Cerebral changes during performance of overlearned arbitrary visuomotor associations. The Journal of neuroscience : the official journal of the Society for Neuroscience. 2006;26(1):117-25.

Hashimoto Y, Sakai KL. Brain activations during conscious self-monitoring of speech production with delayed auditory feedback: an fMRI study. Human brain mapping. 2003;20(1):22-8.

Heim S, Eickhoff SB, Amunts K. Specialisation in Broca's region for semantic, phonological, and syntactic fluency? NeuroImage. 2008;40(3):1362-8.

Hirshorn EA, Thompson-Schill SL. Role of the left inferior frontal gyrus in covert word retrieval: neural correlates of switching during verbal fluency. Neuropsychologia. 2006;44(12):2547-57.

Jones HM, Brammer MJ, O'Toole M, Taylor T, Ohlsen RI, Brown RG, et al. Cortical effects of quetiapine in first-episode schizophrenia: a preliminary functional magnetic resonance imaging study. Biological psychiatry. 2004;56(12):938-42.

Karunanayaka P, Schmithorst VJ, Vannest J, Szaflarski JP, Plante E, Holland SK. A group independent component analysis of covert verb generation in children: a functional magnetic resonance imaging study. NeuroImage. 2010;51(1):472-87.

Kircher T, Krug A, Markov V, Whitney C, Krach S, Zerres K, et al. Genetic variation in the schizophrenia-risk gene neuregulin 1 correlates with brain activation and impaired speech production in a verbal fluency task in healthy individuals. Human brain mapping. 2009;30(10):3406-16.

Kircher T, Nagels A, Kirner-Veselinovic A, Krach S. Neural correlates of rhyming vs. lexical and semantic fluency. Brain research. 2011;1391:71-80.

Kircher T, Whitney C, Krings T, Huber W, Weis S. Hippocampal dysfunction during free word association in male patients with schizophrenia. Schizophrenia research. 2008;101(1-3):242-55.

Kleinhans NM, Muller RA, Cohen DN, Courchesne E. Atypical functional lateralization of language in autism spectrum disorders. Brain research. 2008;1221:115-25.

Kronbichler M, Hutzler F, Staffen W, Mair A, Ladurner G, Wimmer H. Evidence for a dysfunction of left posterior reading areas in German dyslexic readers. Neuropsychologia. 2006;44(10):1822-32.

Lee HJ, Truy E, Mamou G, Sappey-Marinier D, Giraud AL. Visual speech circuits in profound acquired deafness: a possible role for latent multimodal connectivity. Brain : a journal of neurology. 2007;130(Pt 11):2929-41.

Lillywhite LM, Saling MM, Demutska A, Masterton R, Farquharson S, Jackson GD. The neural architecture of discourse compression. Neuropsychologia. 2010;48(4):873-9.

Lopez-Larson MP, Bogorodzki P, Rogowska J, McGlade E, King JB, Terry J, et al. Altered prefrontal and insular cortical thickness in adolescent marijuana users. Behavioural brain research. 2011;220(1):164-72.

Marien P, Baillieux H, De Smet HJ, Engelborghs S, Wilssens I, Paquier P, et al. Cognitive, linguistic and affective disturbances following a right superior cerebellar artery infarction: a case study. Cortex; a journal devoted to the study of the nervous system and behavior. 2009;45(4):527-36.

Markov V, Krug A, Krach S, Whitney C, Eggermann T, Zerres K, et al. Genetic variation in schizophrenia-risk-gene dysbindin 1 modulates brain activation in anterior cingulate cortex and right temporal gyrus during language production in healthy individuals. NeuroImage. 2009;47(4):2016-22.

Mbwana J, Berl MM, Ritzl EK, Rosenberger L, Mayo J, Weinstein S, et al. Limitations to plasticity of language network reorganization in localization related epilepsy. Brain : a journal of neurology. 2009;132(Pt 2):347-56.

Meijer JH, Schmitz N, Nieman DH, Becker HE, van Amelsvoort TA, Dingemans PM, et al. Semantic fluency deficits and reduced grey matter before transition to psychosis: a voxelwise correlational analysis. Psychiatry research. 2011;194(1):1-6.

Nagels A, Kirner-Veselinovic A, Krach S, Kircher T. Neural correlates of S-ketamine induced psychosis during overt continuous verbal fluency. NeuroImage. 2011;54(2):1307-14.

Nosarti C, Shergill SS, Allin MP, Walshe M, Rifkin L, Murray RM, et al. Neural substrates of letter fluency processing in young adults who were born very preterm: alterations in frontal and striatal regions. NeuroImage. 2009;47(4):1904-13.

Papagni SA, Mechelli A, Prata DP, Kambeitz J, Fu CH, Picchioni M, et al. Differential effects of DAAO on regional activation and functional connectivity in schizophrenia, bipolar disorder and controls. NeuroImage. 2011;56(4):2283-91.

Pelletier I, Paquette N, Lepore F, Rouleau I, Sauerwein CH, Rosa C, et al. Language lateralization in individuals with callosal agenesis: an fMRI study. Neuropsychologia. 2011;49(7):1987-95.

Perani D, Abutalebi J, Paulesu E, Brambati S, Scifo P, Cappa SF, et al. The role of age of acquisition and language usage in early, high-proficient bilinguals: an fMRI study during verbal fluency. Human brain mapping. 2003;19(3):170-82.

Polito C, Berti V, Ramat S, Vanzi E, De Cristofaro MT, Pellicano G, et al. Interaction of caudate dopamine depletion and brain metabolic changes with cognitive dysfunction in early Parkinson's disease. Neurobiology of aging. 2012;33(1):206 e29-39.

Porter JN, Collins PF, Muetzel RL, Lim KO, Luciana M. Associations between cortical thickness and verbal fluency in childhood, adolescence, and young adulthood. NeuroImage. 2011;55(4):1865-77.

Powell HW, Parker GJ, Alexander DC, Symms MR, Boulby PA, Wheeler-Kingshott CA, et al. Hemispheric asymmetries in language-related pathways: a combined functional MRI and tractography study. NeuroImage. 2006;32(1):388-99.

Ragland JD, Moelter ST, Bhati MT, Valdez JN, Kohler CG, Siegel SJ, et al. Effect of retrieval effort and switching demand on fMRI activation during semantic word generation in schizophrenia. Schizophrenia research. 2008;99(1-3):312-23.

Rimrodt SL, Clements-Stephens AM, Pugh KR, Courtney SM, Gaur P, Pekar JJ, et al. Functional MRI of sentence comprehension in children with dyslexia: beyond word recognition. Cerebral cortex. 2009;19(2):402-13.

Rimrodt SL, Peterson DJ, Denckla MB, Kaufmann WE, Cutting LE. White matter microstructural differences linked to left perisylvian language network in children with dyslexia. Cortex; a journal devoted to the study of the nervous system and behavior. 2010;46(6):739-49.

Rodda J, Dannhauser T, Cutinha DJ, Shergill SS, Walker Z. Subjective cognitive impairment: functional MRI during a divided attention task. European psychiatry : the journal of the Association of European Psychiatrists. 2011;26(7):457-62.

Salmon E, Perani D, Herholz K, Marique P, Kalbe E, Holthoff V, et al. Neural correlates of anosognosia for cognitive impairment in Alzheimer's disease. Human brain mapping. 2006;27(7):588-97.

Scott FE, Mechelli A, Allin MP, Walshe M, Rifkin L, Murray RM, et al. Very preterm adolescents show gender-dependent alteration of the structural brain correlates of spelling abilities. Neuropsychologia. 2011;49(9):2685-93.

Shah C, Erhard K, Ortheil HJ, Kaza E, Kessler C, Lotze M. Neural correlates of creative writing: an fMRI study. Human brain mapping. 2013;34(5):1088-101.

Sommer IE, Diederen KM, Blom JD, Willems A, Kushan L, Slotema K, et al. Auditory verbal hallucinations predominantly activate the right inferior frontal area. Brain : a journal of neurology. 2008;131(Pt 12):3169-77.

St Jacques PL, Rubin DC, Cabeza R. Age-related effects on the neural correlates of autobiographical memory retrieval. Neurobiology of aging. 2012;33(7):1298-310.

Tanaka H, Arai M, Harada M, Hozumi A, Hirata K. Cognition and event-related potentials in adult-onset non-demented myotonic dystrophy type 1. Clinical neurophysiology : official journal of the International Federation of Clinical Neurophysiology. 2012;123(2):261-9.

Van Ettinger-Veenstra H, Ragnehed M, McAllister A, Lundberg P, Engstrom M. Right-hemispheric cortical contributions to language ability in healthy adults. Brain and language. 2012;120(3):395-400.

Viskontas IV, Boxer AL, Fesenko J, Matlin A, Heuer HW, Mirsky J, et al. Visual search patterns in semantic dementia show paradoxical facilitation of binding processes. Neuropsychologia. 2011;49(3):468-78.

Vitali P, Abutalebi J, Tettamanti M, Rowe J, Scifo P, Fazio F, et al. Generating animal and tool names: an fMRI study of effective connectivity. Brain and language. 2005;93(1):32-45.

Voets NL, Adcock JE, Flitney DE, Behrens TE, Hart Y, Stacey R, et al. Distinct right frontal lobe activation in language processing following left hemisphere injury. Brain : a journal of neurology. 2006;129(Pt 3):754-66.

Volz KG, Schooler LJ, von Cramon DY. It just felt right: the neural correlates of the fluency heuristic. Consciousness and cognition. 2010;19(3):829-37.

Voss JL, Federmeier KD, Paller KA. The potato chip really does look like Elvis! Neural hallmarks of conceptual processing associated with finding novel shapes subjectively meaningful. Cerebral cortex. 2012;22(10):2354-64.

Weems SA, Reggia JA. Simulating single word processing in the classic aphasia syndromes based on the Wernicke-Lichtheim-Geschwind theory. Brain and language. 2006;98(3):291-309.

Weiss EM, Hofer A, Golaszewski S, Siedentopf C, Felber S, Fleischhacker WW. Language lateralization in unmedicated patients during an acute episode of schizophrenia: a functional MRI study. Psychiatry research. 2006;146(2):185-90.

Whalley HC, Sussmann JE, Chakirova G, Mukerjee P, Peel A, McKirdy J, et al. The neural basis of familial risk and temperamental variation in individuals at high risk of bipolar disorder. Biological psychiatry. 2011;70(4):343-9.

You H, Gaab N, Wei N, Cheng-Lai A, Wang Z, Jian J, et al. Neural deficits in second language reading: fMRI evidence from Chinese children with English reading impairment. NeuroImage. 2011;57(3):760-70.

Ziemus B, Baumann O, Luerding R, Schlosser R, Schuierer G, Bogdahn U, et al. Impaired working-memory after cerebellar infarcts paralleled by changes in BOLD signal of a cortico-cerebellar circuit. Neuropsychologia. 2007;45(9):2016-24.
